# Two genes, one culprit - a functional candidate validation of a *SPATA7* deletion in dogs with day blindness/retinal degeneration

**DOI:** 10.1371/journal.pgen.1011961

**Published:** 2025-12-01

**Authors:** Leonardo Murgiano, Jessica K. Niggel, Kei Takahashi, Valérie L. Dufour, Catharina R. Grubaugh, Raghavi Sudharsan, Jennifer C. Kwok, Doreen Becker, Esha Banerjee, Wen-Mei Yu, Tosso Leeb, Cheng-Kui Qu, William A. Beltran, Gustavo D. Aguirre

**Affiliations:** 1 Division of Experimental Retinal Therapies, Department of Clinical Sciences & Advanced Medicine, School of Veterinary Medicine, University of Pennsylvania, Philadelphia, United States of America; 2 Sylvia M. Van Sloun Laboratory for Canine Genomic Analysis, School of Veterinary Medicine, University of Pennsylvania, Philadelphia, Pennsylvania, United States of America; 3 School of Dental Medicine, University of Pennsylvania, Philadelphia, Pennsylvania, United States of America; 4 Research Institute for Farm Animal Biology (FBN), Working group Genome Annotation, Dummerstorf, Mecklenburg-Vorpommern, Germany; 5 Comparative Pathology Core (CPC), Department of Pathobiology, School of Veterinary Medicine, University of Pennsylvania, Philadelphia, Pennsylvania, United States of America; 6 Department of Pediatrics, Aflac Cancer and Blood Disorders Center, Winship Cancer Institute, Children’s Healthcare of Atlanta, Emory University School of Medicine, Atlanta, Georgia, United States of America; 7 Institute of Genetics, Vetsuisse Faculty, University of Bern, Bern, Switzerland; Pennsylvania State University, UNITED STATES OF AMERICA

## Abstract

Inherited retinal diseases (IRDs) are a diverse group of disorders that share common vision deficits ranging from early onset blindness to severe and progressive later-onset disease. We report a form of early-onset day-vision loss, cone-rod dystrophy, in the Standard poodle. Through GWAS and homozygosity mapping, a large deletion on CFA8:NC_049229.1:g.60,022,583_60,040,453del was found which removes 3’ portions of two different genes, *PTPN21* and *SPATA7*, presenting a challenge for assessing the actual causative gene in a multi-gene large deletion. All affected dogs were homozygous for the mutant allele, which segregated perfectly with the phenotype within the breed. The variant was absent in 1879 dogs from the Dog10K database. While the role of *SPATA7* for retinal disease has been established in human patients and genetically engineered mice, the role of *PTPN21* in the retina is unclear even though it is expressed in rod and cone photoreceptors. Expression of whole and truncated transcripts for both genes was detected in skin fibroblasts from controls and cases. Retinal RNA analysis of *PTPN21* splicing suggests that at least one unmodified transcript is still present in mutants. *Ptpn21*-/- knockout mice did not have an ocular phenotype, and IHC for rod- and cone-specific opsins detected no cone or rod abnormalities suggesting that PTPN21 loss has minimal to no contributory role towards the retinal phenotype in mutants. The variant leads to a deletion of the 3’-end of the *SPATA7* transcript: XM_038545497.1:r.1,314_1,629delins[g.60,018,954–60,018,990], p.(XP_038401425.1: Asp361GlufsTer2), reducing the predicted protein from 595 to 361 AA. Ultrastructure expansion microscopy (U-ExM) enabled the detection of a distinct SPATA7 signal around the transition zone of the primary cilium in photoreceptors and fibroblasts of WT dogs, which was absent in affected dog. We posit that SPATA7 deficiency is the main cause of the condition, and propose this disease as a model for the *SPATA7*-related form of cone-rod dystrophy in humans. Our work shows an example of functional refinement of a multi-gene deletion variant using a multi-technique approach.

## Author summary

Inherited retinal diseases are a diverse group of genetic conditions that cause blindness. Like humans, dogs are also affected by retinal diseases, with many being reported and studied. The mapping and characterization of canine inherited conditions is essential for the health of affected breeds, and, as models for human conditions, canine retinal diseases can also provide new insights into phenotype, gene function, biological mechanisms and treatments. Using genome-wide association and homozygosity mapping combined with whole genome sequencing, we found a large-sized genetic variant in Standard poodle that partially deletes two genes, *PTPN21* and *SPATA7*. Through gene expression, transcript analysis of both genes, retinal single-cell RNA analysis, ultrastructure expansion microscopy, and a *Ptpn21* knockout murine model, we show that *SPATA7* is the likely disease candidate. We propose this canine model for the *SPATA7*-related form of cone-rod dystrophy in humans. Our work shows an example of functional refinement of a deletion variant affecting more than one gene, using a multi-angled approach involving an array of diverse techniques.

## Introduction

Since the early part of this century there has been a virtual explosion of genetic and genomic information and resources, including canine studies. The dog was the first non-primate and non-rodent mammal whose genome sequence was published [1] and was subsequently used as a model organism for genomic studies [2–4]. Once the first linkage map of the dog genome was published in 1997 [5], the rapid identification of the first autosomal retinal disorder locus of dogs, progressive rod-cone degeneration (prcd), soon followed [6]. Currently, a wealth of genomic tools is available. The study of inherited retinal diseases has been leading the way in developing the needed tools and resources [1,7], and now more genes/and genetic variants of canine retina have been identified than for any other organ system [8–12].

Retinal diseases in dogs can be isolated disorders, usually affecting the retinal pigment epithelium (RPE) and/or the photoreceptors - either rods and/or cones (selected examples include [6,10,13–17]. In other cases, the diseases are syndromic and associated with oculo-skeletal defects [18] or neurological abnormalities [19,20]. The progress made in identifying many different inherited retinal diseases and their causative genes/mutations in dogs has created confusion on how these diseases are named and classified. To address this issue a recent publication has proposed consensus guidelines for nomenclature for the retinal diseases affecting dogs and other companion animals [10]. Like many other inherited genetic conditions, canine ocular diseases have been associated with different types of genetic variants ranging from single base pair changes to large structural variants [8–10].

By far the largest group of inherited retinal diseases in dogs falls under the progressive retinal atrophy (PRA) rubric. Although caused by many non-allelic pathogenic variants, these diseases share generally similar clinical signs, e.g., appearance of alterations in the retinal fundus, eventual blindness and secondary cataracts. The diseases vary in terms of age of onset, the rate of progression, the type and degree of visual impairment, and, when analyzed, the retinal functional and structural abnormalities [9,12]. Usually, PRA is characterized by initial night blindness followed by day vision impairment and eventual blindness that is dependent on the specific breed and/or variant (for example see Aguirre, 1998, Zangerl 2006 [16,21]). However, a sub group of PRAs [10], the cone-rod dystrophies, have vision deficits first in daylight followed by night blindness. In dogs, variants in at least 4 genes are responsible for the cone-rod dystrophy phenotype: *ADAM9* [22] *NPHP4* [23], *NPHP5* [24] and *RPGRIP1* [25]. In this study we present the mapping and gene identification of a new severe early onset cone-rod dystrophy in the Standard poodle (SP) breed which we refer to as Day Blindness/Retinal Degeneration (DB/RD). The disease is associated with a large deletion affecting two genes, but functional candidate validation studies detailed in this manuscript establish *SPATA7* as causal to the disease.

## Results

### Phenotype

**Fig 1:**
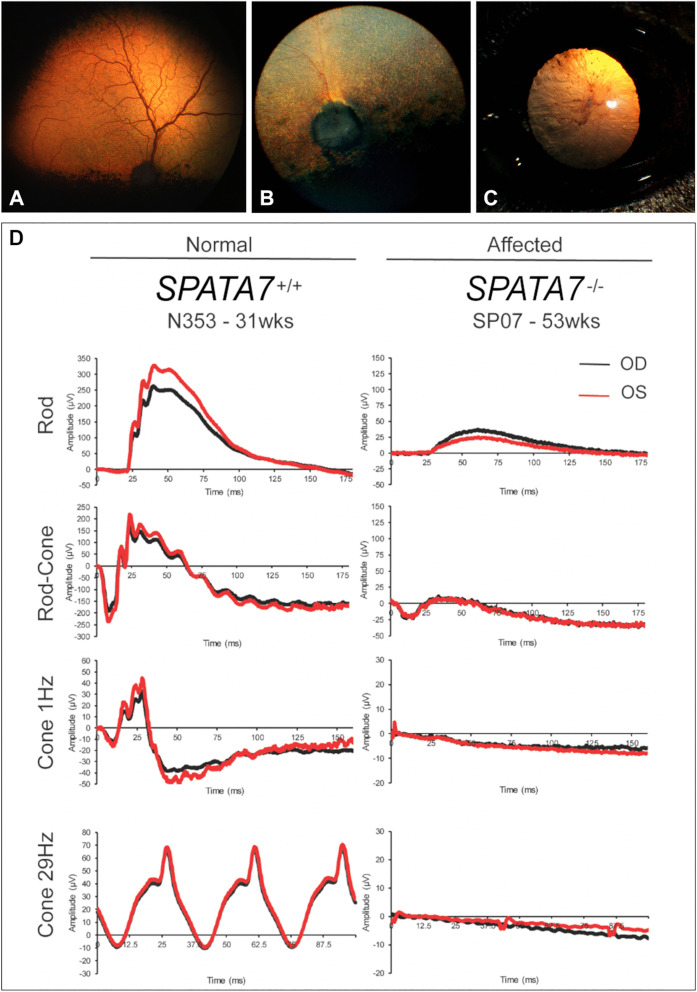
Retinal disease phenotype. A-D: Fundus findings in *SPATA7*-affected dogs. **(A)** Right eye of SP07 at 1 year of age. The retinal vessels are normal and there is no generalized increase in reflectivity from the tapetum at this stage of the disease. **(B)** Right eye of SP20 at 2.7 years of age. There is diffuse retinal hyperreflectivity (Note: light intensity used for the photograph is 10 times less intense than that used for a normal eye) with marked attenuation and loss of retinal vessels, and optic nerve head atrophy. **(C)** Left eye of SP20 at 7.4 years. Secondary cataracts are developing; shown is the anterior cortical cataract extending from the nasal quadrant. **(D)**: Electroretinograms recorded from a normal and affected (SP07) dog. The affected dog has absent cone responses, and rod responses are abnormally low in amplitude. OD = right eye; OS = left eye. Note that the vertical scales for amplitude are different because the amplitudes of the affected dog are much lower.

Affected dogs had severe vision deficits present at a young age, generally before 2–3 months of age, and characterized by very poor to absent vision under photopic conditions, but with no evidence of photophobia. Grossly, vision under scotopic conditions was initially normal, but was not critically assessed, and worsened over time; in older animals, blindness developed but onset was variable. For example, dog SP07 (Table 1) has remained completely day blind since first diagnosed at 3–4 months of age, and by 3.3 years of age, he still had functional vision in a regular home environment. At that time, there was no evidence of further progressive degenerative retinal changes by indirect ophthalmoscopy from those recorded at 1 year of age (Fig 1A). In contrast, dog SP20 (Table 1) had severe vision impairment/blindness under all lighting conditions by 2.7 years of age, along with end-stage retinal degeneration visible by indirect ophthalmoscopy; and cortical cataracts were distinct after more than 7 years of age (Fig 1B and 1C).

**Table 1:** Genotyped Standard poodle samples for the deletion. Age at time of clinical diagnosis by board-certified veterinary ophthalmologists who confirmed the presence of a severe vision deficit is included. Dogs used for the initial SNP-based mapping are underscored and in italics. The large family tree is reported in S2 Fig and shows cases whose ancestry could be reconstructed.

Diagnosis ID	sex	Present on pedigree	Clinical diagnosis age	Genetic test Result
*SP03*	f	Yes	1y0m	Homozygous mutant
*SP04*	f	No	0y9m	Homozygous mutant
*SP05*	f	Yes	0y2m	Homozygous mutant
*SP06*	f	No	not available	Homozygous mutant
*SP07*	m	Yes	0y4m	Homozygous mutant
SP08	f	No	0y6m	Homozygous mutant
SP09	f	Yes	0y10m	Homozygous mutant
SP10	m	Yes	not available	Homozygous mutant
SP11	f	Yes	not available	Homozygous mutant
*SP12*	m	Yes	0y5m	Homozygous mutant
SP13	m	No	0y4m	Homozygous mutant
SP14	f	No	0y10m	Homozygous mutant
SP15	f	Yes	not affected	Heterozygous
SP16	f	Yes	<10 wks	Homozygous mutant
SP17	m	Yes	<10 wks	Homozygous mutant
SP18	f	Yes	not available	Homozygous mutant
SP19	m	Yes	0y3m	Homozygous mutant
SP20	f	Yes	3y8m	Homozygous mutant
SP21	f	No	0y11m	Homozygous mutant
SP22	m	No	1y1m	Homozygous mutant
SP23	m	Yes	0y3m	Homozygous mutant
SP24	m	Yes	1y7m	Homozygous mutant
SP29	m	No	0y3m	Homozygous mutant
GD24	f	Yes	not available	Homozygous mutant

Full-field electroretinography (ERG) was used for objective assessment of retinal function. Based on submitted reports, and our testing of a subset of dogs included in the study, cone ERG responses were absent as early as 7 weeks of age. We also found that as early as 15 weeks of age, rod responses were reduced by ~60–80% in dogs that showed no vascular attenuation or generalized hyperreflectivity (Fig 1D; note the ERG recordings of SP07 were done within 1 week of the retinal examination shown in Fig 1A). Over time, rod responses were further decreased and no longer recordable, and the ERG was considered ‘extinguished’.

### Pedigree and selection of study animals

A total of six cases, one male and five females, were initially collected. Information on the immediate family of the cases indicated unaffected parents and unaffected siblings (S1A Fig). Additionally, pedigree material was gathered on the available ancestry of the cases. Together with pedigree records from archived samples a full pedigree of the whole case group could be constructed. Interestingly, a shared ancestor born in 1923 was found. The complete tree, including other cases whose ancestry information we had access to, is shown in S2 Fig. The available family history information, showing no phenotypically affected parents and mixed litters of affected and unaffected males and females alike, suggested an autosomal recessive mode of inheritance with the possibility of a shared genetic etiology. Therefore, we considered X-linked or autosomal dominant inheritance unlikely.

### SNP genotyping and mapping

**Fig 2:**
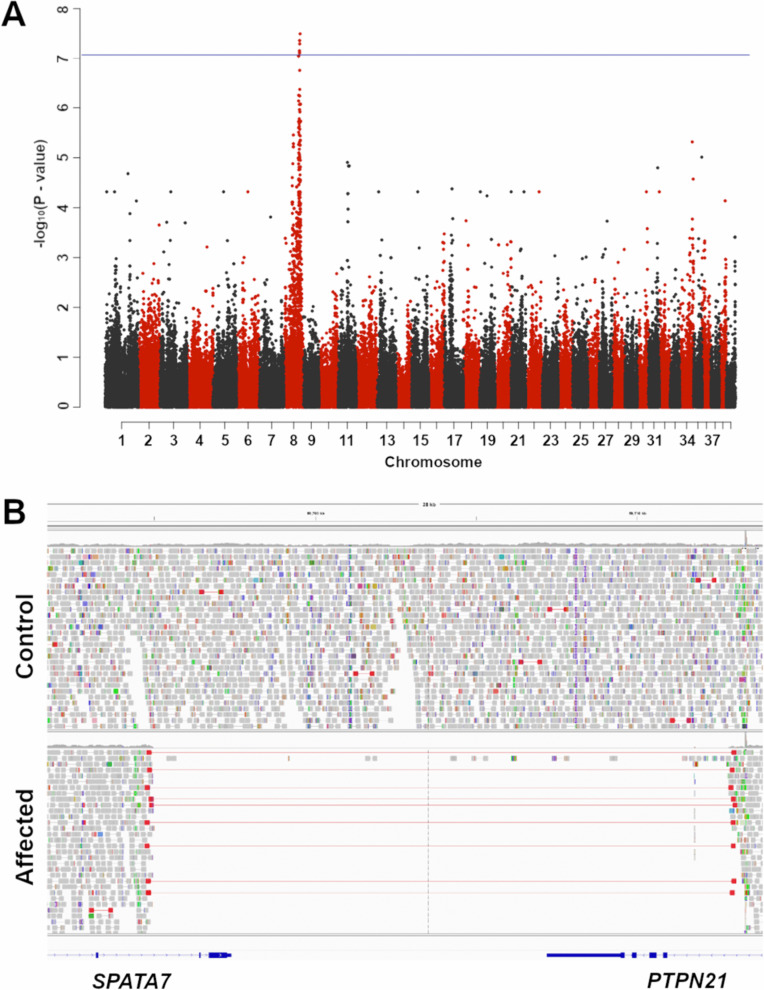
Genome-wide association analysis and candidate causative variant. **(A)** Results of the genome-wide association study (GWAS) obtained from analyzing the Illumina SNP microarray data showing the negative log of the raw p-values calculated with the genotypic association test (max -log p-value = 7.52), marker BICF2G630416220). Five SNPs were above the Bonferroni correction threshold (blue horizontal line). **(B)** Representative whole-genome sequencing reads in the region of the 17.8 kb-spanning candidate causative variant visualized with IGV. A homozygous wild-type control (*top*) displays normal coverage for the interval, while a case (SP03) shows a lack of coverage for this interval. Additionally, the mapping visualized in “show paired” mode shows several reads, colored in red, whose pairs map at the two ends of the gap. Note that the 3’ ends of the *SPATA7* (two predicted exons) and *PTPN21* (four predicted exons) genes are missing in the affected dog.

A combined GWAS and homozygosity mapping approach was used to map the critical interval containing the causative variant. Initially, six cases and 46 controls, including two unaffected siblings, were genotyped on Illumina 220k or 170k canine SNP arrays (see Methods). GWAS quality control filtered the markers down to 149,064 informative markers for mapping; the calculated lambda was 1.52 indicating significant level of stratification in the selected sample pool, and a mixed-model was applied for the GWAS. The analysis outlined a significant peak on chromosome 8 (CFA8) (Figs 2A and S3A). The top 50 + associated markers were all on CFA8. Additionally, 5 of these SNPs were above the very strict Bonferroni correction threshold, suggesting a very strong association. Of note, the QQ-plot did show a significant degree of inflation even after adjustment (S3A Fig).

Subsequently, the candidate variant was fine-mapped using homozygosity mapping on the six genotyped cases. With the assumption of a monogenic recessive inheritance based on the observed disease segregation, extended regions of homozygosity were examined with the expectation that the disease allele and flanking chromosomal segments (~1 Mb or higher) in the affected dogs would be homozygous identical by descent (IBD). This initial filtering indicated the 6 cases sharing two regions (canFam4 coordinates): one on CFA8 (markers BICF2G630415989-BICF2G630416371; chr8: 59,617,725–60,566,231), and a region on CFA21 (markers BICF2G630649945-TIGRP2P281318_rs9013981; chr21: 23,880,170–24,289,233). No other region was shared by all the cases. Results for homozygosity mapping are shown in S1B Fig, highlighting the very small number of shared regions for the sample pool, along with their small size, approximately on the lower end of our threshold. This suggested that the candidate interval to be considered was UU_Cfam_GSD_1.0 (canFam4): CFA8:59,617,725–60,566,231 (S3B and S3C Fig). The best associated markers from the GWAS were located outside of the shared homozygous interval on chromosome 8 by at least 0.5 Mb. This is not unusual in canine GWAS and is most likely driven by the long within-breed linkage disequilibrium and recombination events in control animals or their ancestors.

### Variant detection and genotyping

Whole genome sequencing was carried out for case SP03, reads were mapped against the UU_Cfam_GSD_1.0 reference (canFam4) resulting in an average coverage of 28.49x. A total of 1,269 variants (SNVs and small indels) were called in the CFA 8 critical interval, a region including eight genes. Comparing these against the Dog10K database [26,27] and European Variant Database (EVA, https://www.ebi.ac.uk/eva/) eliminated every called SNV and small indel variant in the interval as a potential candidate. Large structural variants were then analyzed and filtered against the UU_Cfam_GSD_1.0 (canFam4) Dog10K database. A single structural variant was exclusively identified in the affected dog sequenced for the study: a homozygous deletion designated as NC_049229.1:g.60,022,583_60,040,453del (Fig 2B).

This ~17.8 kb deletion was visually verified through the Integrative Genomics Viewer and encompasses the 3’-ends of two opposed genes, *SPATA7* and *PTPN21*. The deletion involves the last two exons of *SPATA7* (exons 11 and 12) and the last four coding exons of *PTPN21* (exons 15–18), henceforth named *[SPATA7/PTPN21]DEL* (Fig 2B). Loss-of-function variants in *SPATA7* encoding spermatogenesis-associated protein 7 are responsible for human Leber Congenital Amaurosis, and Retinitis Pigmentosa [28]. *PTPN21* belongs to the PTP group of genes known to have a signaling role and to regulate a variety of cellular processes including cell growth, differentiation, mitotic cycle, motility, deformability, and oncogenic transformation; to the authors’ knowledge, variants in *PTPN21* have not been associated with retinal diseases.

As the *[SPATA7/PTPN21]DEL* variant deleted coding regions of two genes, one linked to retinal disease, we considered it a very likely putative causative variant. To this end, a PCR test based on 3 primers was developed (see Materials and Methods, and S3C Fig). The variant was then genotyped on additional cases including an affected Goldendoodle (Golden retriever and Standard poodle intercross/backcross) as well as controls, and perfect genotype-phenotype association was observed (Table 2).

**Table 2:** Distribution of the *[SPATA7/PTPN21]DEL* variant in Standard poodles and in a database of canine variants. The 22 SP dogs and the Goldendoodle are a total of those collected and genotyped as described in Methods, including dogs initially SNP genotyped and whole genome sequenced as well as additional cases collected after the mapping of the proposed variant. The “other breeds” line consists in the manta-called vcf file in the Dog10K database (no retinal phenotype verified, genotype detected by Manta).

Breed and availability of retinal phenotype	N of dogs	*[SPATA7/PTPN21]DEL*		
		wt/wt	wt/DEL	DEL/DEL
Standard poodle – Cases	22	0	0	22
Goldendoodle – Case	1	0	0	1
Standard poodle – Unaffected	63	42	21	0
Other breeds (Dog10K)*	1879	1879	0	0
total	1965	1921	21	23

*database; dogs not genotyped by PCR

The Gandolfo method to estimate the age of the mutation using the online tool created by their group (see Methods) was used. The analysis suggested that the mutant allele arose 22.6 generations ago, with a 95% confidence interval of 10.8 - 34.2 generations. This result is consistent with the putative shared ancestor identified as being born in 1923 (S2 Fig) which shows 22–35 generations between the case used for SNP genotyping and the putative common ancestor, and in which several ancestors are bred with dogs down the line.

### Expression of *PTPN21* and *SPATA7*

**Fig 3:**
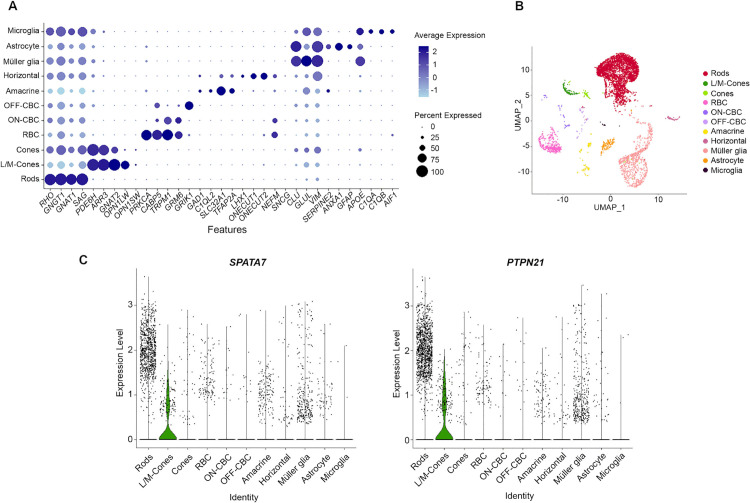
Single-cell RNA sequencing analysis of *PTPN21* and *SPATA7* expression in the retinas of two normal adult dogs (N350 and N354) at 40 weeks of age. **(A)** Dot plot showing selected marker genes used to identify major retinal cell clusters. **(B)** UMAP plot depicting the distribution of identified retinal cell types. **(C)** Violin plots illustrating expression of *SPATA7* and *PTPN21* across retinal cell types. Expression of both genes was notably higher in rod photoreceptors but was restricted to a small subset, as indicated by the narrow yet pronounced peaks in the rod clusters. In contrast, a broader distribution of expression was observed in cone photoreceptors, suggesting that a greater proportion of cone cells express these genes.

To identify the retinal cell populations expressing *PTPN21* and *SPATA7*, single-cell RNA-seq datasets generated in our lab from two normal adult dogs were examined. Both *PTPN21* and *SPATA7* showed distinctive expression patterns in different retinal cell types. The expression levels were notably higher in rods, but the expression was limited to a small subset of rods. In cones, the expression levels of both genes were generally lower compared to the rods, suggesting moderate expression across a larger cone population. Additionally, both genes exhibited low-level expression in a few cells from other clusters, including Müller glia and amacrine cells. Overall, *PTPN21* and *SPATA7* were found to be predominantly expressed in cones in the normal adult canine retina (Fig 3).

**Fig 4:**
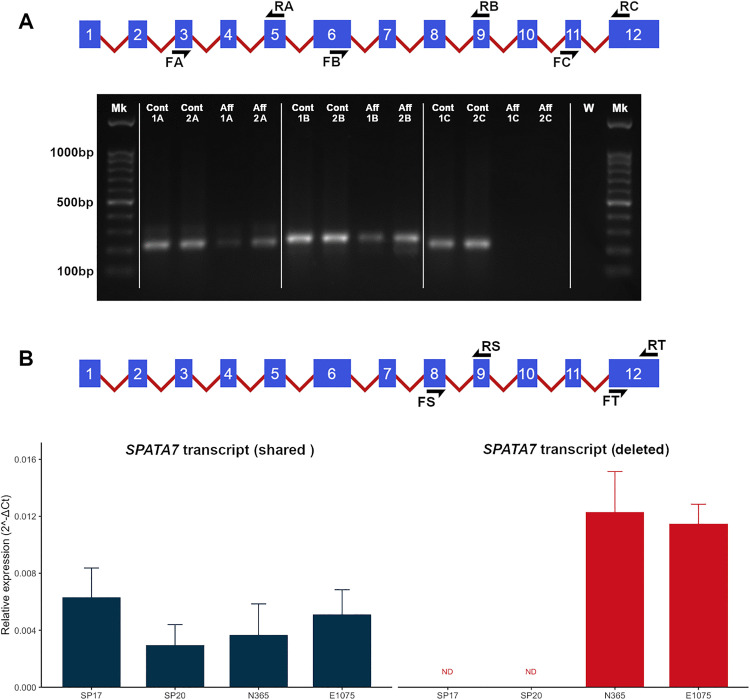
Expression of the *SPATA7* transcript in cultured fibroblasts. **(A)** Top: Schematic of the *SPATA7* transcript showing the location of primers used to amplify amplicon A (exons 3-5, 230 bp), amplicon B (exons 6-9, 252 bp), and amplicon C (exons 11-12, 221 bp). Bottom: Products of PCR on cDNA from two fibroblast cultures (“1” and “2”) derived from one control (“Cont”) and one affected (“Aff”) dog showing the absence of amplicon C (exons 11-12) in the affected dog. W: water control. Mk: ladder. **(B)** Schematic of the *SPATA7* transcript showing the location of primers used for real-time PCR (shared: 99 bp, Exon 8 to 9; deleted in affected: 99 bp, Exon 12) (top). Mean ΔCt values (±SEM or standard deviation) of amplification from cDNA from cultured fibroblasts derived from control (N365 and E1075) and affected (SP17 and SP20) dogs relative to *GAPDH* (bottom). PCRs were performed in triplicate for each sample. ΔCt values were compared between pooled affected SPs (SP17 + SP20) and pooled controls (N365 + E1075) using Welch’s t-test. There was no significant difference (SPs/Others = 1.00, 95% CI 0.57–1.76; p = 0.992). Note that the higher value of the deleted region amplicon exclusive to the controls is due to higher efficiency of that reaction compared to the shared Exons 8 and 9 target. ND = not detectable.

To determine the impact of the *[SPATA7/PTPN21]DEL* variant, RNA was extracted from control and mutant skin fibroblasts as affected retinal tissues were not available in replicates. Separate fibroblast cultures were done from skin biopsies of a genotyped case (SP12, see Fig 4A), RNA was extracted, and cDNA synthesized (in duplicate from two different fibroblast cultures, also see S4 Fig). The same was carried out on cultured skin fibroblasts of two different canine controls. Initially, standard PCR amplifications targeted at regions of the transcript not affected by the deletion were successful using both case- and control-derived cDNA; targeting exons in the deleted region only resulted in amplicons from control-derived cDNA (detailed in Fig 4A). To assess on whether the mutant transcript was detectable in amounts comparable to the WT, we ran a semi-quantitative (real-time) PCR using RNA from fibroblast from two cases and two controls dogs. Two different reactions targeted a region in the transcript shared by WT and mutant, and the region lost in the mutant. Amplification of the shared region of the transcript (two biological, three technical replicates each) did not show any significant difference in expression between the WT and mutant dogs (Fig 4B).

Analysis of the expression pattern of *PTPN21* in normal canine retina indicated that *PTPN21* has isoforms with alternate splicing suggesting a split of the whole transcript at the level of exon 12, and the variant we describe leaves at least one of the *PTPN21* transcripts (each one coding for a different active domain) intact (see S1 File).

The results indicate that the mutated *SPATA7* transcript is truncated and not subjected to nonsense-mediated decay. Careful observation of the RNA-seq data of the case we generated, shows absence of splicing of Exon10 and the mutant transcript ending after exon 9 (S4 Fig and S2 File). The variant leads to a deletion of the 3’-end of the *SPATA7* transcript: XM_038545497.1:r. 1,314_1,629delins[g.60,018,954–60,018,990]. This results into a substitution of an Asp into a Glu in position 361 and a premature stop codon immediately thereafter p.(XP_038401425.1: Asp361GlufsTer2), reducing the predicted protein from 595 to 361 AA (total loss 40% of the protein.

### Immunocytochemistry of canine retina and fibroblasts

**Fig 5:**
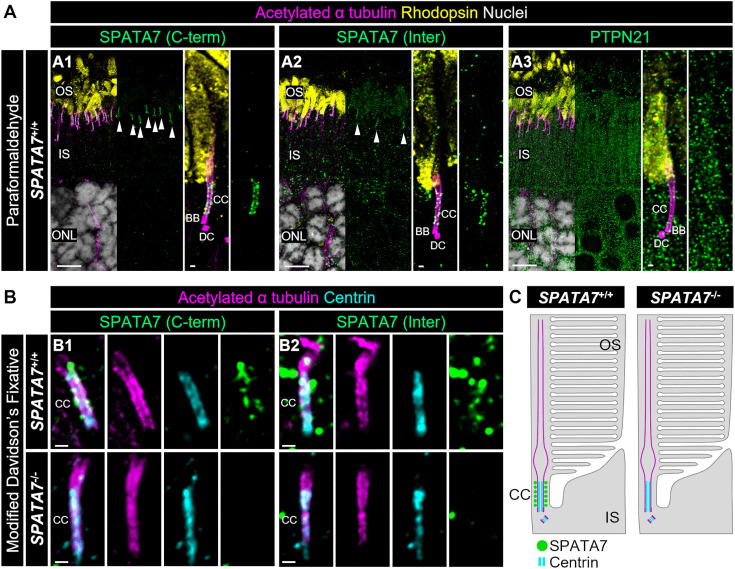
Staining of endogenous SPATA7 and PTPN21 in canine retina using U-ExM. **(A)** Confocal images of paraformaldehyde-fixed adult control (*SPATA7*^*+/+*^) retina processed by U-ExM and IHC for the C-terminal region of SPATA7 (green, A1), the internal region of SPATA7 (green, A2), and PTPN21 (green, A3). The ciliary axoneme and rod outer segment were visualized by labeling with acetylated α-tubulin (magenta) and rhodopsin (yellow), respectively. White arrowheads in the widefield views (left) indicate the specific SPATA7 signal pattern along the CC. The right panels show highly magnified views of individual rod photoreceptors. While the SPATA7 is located in the ciliary axoneme (A1,2), PTPN21 is not (A3). **(B)** Immunolabeling for the C-terminal (green, B1) and internal (green, B2) regions of SPATA7 in *SPATA7*^*+/+*^ (upper panels) and *SPATA7*^*-/-*^ (lower panels) retinas fixed with Modified Davidson’s Fixative. The CC was visualized using centrin, a scaffolding protein of the CC inner structure (cyan). SPATA7 is located in the connecting cilium of normal retina, but not in the mutant. **(C)** Schematic representation illustrating the localization and absence of SPATA7 protein in the CC of *SPATA7*^*+/+*^ (left) and *SPATA7*^*-/-*^ (right) retina, respectively. Scale bars: 5 μm (widefield view) and 200 nm (high-magnification view), after correction for expansion factor. BB, basal body; CC, connecting cilium; DC, daughter centriole; IS, inner segment; ONL, outer nuclear layer; OS, outer segment.

Retinal tissue from one case (SP29) and one control (EM540) were collected; we also used fibroblast cilia as an additional surrogate system for the sensory cilium of photoreceptors. The aim was to assess whether the subcellular localization of endogenous SPATA7 and PTPN21 is influenced by the variant. Immunocytochemistry following induction of ciliogenesis of canine skin fibroblasts (CSFs) derived from affected and control dogs failed to detect specific signals of SPATA7 protein by conventional immunocytochemistry with two different antibodies targeting epitopes that are not present in truncated protein (SPATA7 (Inter) or or partially included in the predicted, truncated protein (SPATA7 (C-term) (retinal results in Fig 5; fibroblasts in S5 Fig; antibodies epitope sequences in Section C in S2 File). Subsequently, we used U-ExM [29], to identify endogenous SPATA7 in retina and fibroblast cilia. SPATA7 exhibited localization in the connecting cilium in retina (Fig 5) and near the transition zone above the mother centriole in fibroblast primary cilia (S5 Fig), consistent with previous findings [30]. In contrast, the SPATA7 signal was completely absent in the mutant retinal cilium, and in the transition zone in the cilia in affected dog fibroblasts (S5 Fig). In fibroblasts, PTPN21 signals were observed in the cytoplasm, particularly pronounced at the base of the primary cilia in both genotypes under non-expanded condition. However, U-ExM samples failed to reveal PTPN21 localization specifically to the primary cilia. Nonetheless, no significant differences in the expression pattern of PTPN21 were observed between cells from affected and control dogs.

### *Ptpn21* knockout mouse

**Fig 6:**
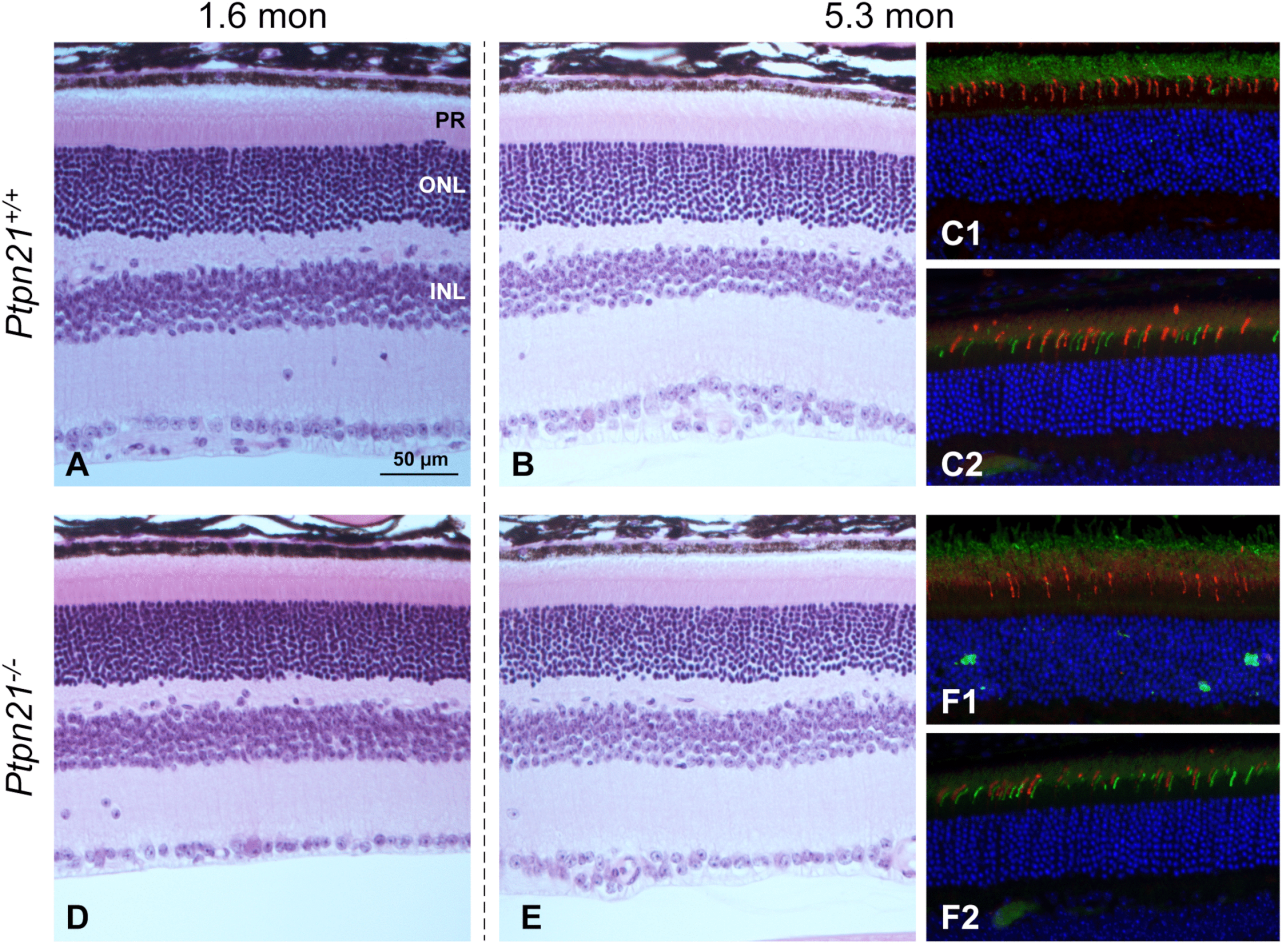
H&E-stained paraffin-embedded (A, B, D, E) or immunolabeled (C1_,_ C2, F1, F2) murine retinal sections from wild-type mice (*Ptpn21*^*+*^^*/*^^*+*^) and homozygous *Ptpn21*^*-/-*^KO mice. Each pair, 1.6 and 5.3 months of age, were collected after euthanasia, and fixed in modified Davidson’s solution. In both wild-type and KO, and at both time points, no observable abnormalities in the retinal structure were detected. In both the wild-type and KO mice, there is normal expression and localization of rod (green) and M-cone (green) opsins (C1, F1) or M- (red) and S- (green) cone opsins (C2_,_ F2). Distribution of the different M- and S-cone classes varies by topography and orientation of the sections [68]. Calibration scale (50µm) applies to all sections.

To investigate a potential contributory role of *PTPN21* on the retinal disease phenotype, we analyzed *Ptpn21*^*-/-*^ knockout (KO) mice that were previously generated by deleting exons 3–10 through a conventional gene targeting approach, resulting in a *Ptpn21* null allele [31]. These mice show undetectable *Ptpn21* mRNA and display defective retention of hematopoietic stem cells in in the niche due to cell mechanical defects [31]. Our macroscopic examination revealed that the eyes of these mice were fully formed and that all intraocular structures were normal. Microscopic examination of the retinas of young (1.6 months) and older (5.3 months) *Ptpn21*^*-/-*^ KO mice showed no retinal abnormalities (Fig 6A, 6B, 6D, and 6F). In addition, we carried out IHC for rod- and cone- (L/M and S-cone) specific opsins in ocular tissue sections from *Ptpn21*^*+/+*^ and *Ptpn21*^*-/-*^ KO mice. The expression and localization of the tested proteins were normal (Fig 6, C1, C2, F1, F2).

## Discussion

In the present study we describe a new autosomal recessive retinal disorder in the SP. Affected dogs show severe cone visual system impairment accompanied by a less severe and more slowly progressive dysfunction and degeneration of the rod cells. The condition, previously referred to as Day Blindness/Retinal Degeneration, is a cone-rod dystrophy that could be included under the Leber congenital amaurosis (LCA) rubric as it causes severe visual impairment or blindness soon after birth.

The combined effort of genealogy reconstruction, precise phenotyping, genetic mapping, and whole genome sequencing along with the use of canine variant databases helped to detect a large structural variant, a deletion removing the 3’-parts of two different genes, *SPATA7* and *PTPN21*, both of which are expressed in the retina. The variant lies in a relatively old and consequently small shared haplotype in the SNP-genotyped cases. scRNA-seq analysis of normal canine retinas showed that both genes have a similar pattern of expression in cones, with at least one *PTPN21* transcript being unaffected by the deletion. In this study, we focused on determining how the two genes, alone or in combination, are responsible for the disease. In inherited disorders involving large structural variants, such as large deletions, it is not always immediately clear whether all the genes involved are all equally responsible for the phenotype [32,33]. Retinal degeneration and other ocular conditions associated with a large deletion have been reported in both humans and dogs, but are uncommon [15,34,35].

Because the variant affected two genes expressed in canine retina, it was necessary to determine if *SPATA7* and/or *PTPN21* were responsible for the disease phenotype. The *Ptpn21*^*-/-*^ KO mouse [31] excluded association of *Ptpn21* with the retinal disease. In contrast, the causative role of *SPATA7* in human retinopathies is well established [28,36–39], as its role in the photoreceptor connecting cilium [30,40]. Of significance is that *Spata7*^*-/-*^ KO mice have a severe retinal phenotype [41] while the present study established that the retina and photoreceptors of *Ptpn21*^*-/-*^ KO mice are structurally normal.

*SPATA7* is evolutionarily conserved in mammals and encodes a protein mostly expressed in spermatocytes and retina. The gene was first identified in spermatocytes, as a gene with a differential expression in specific stages of spermatogenesis [42,43]. The identified human transcripts show an alternate splicing of exon 3; transcripts including this exon are more abundantly expressed in neuronal tissues such as cerebellum, and retina, while the variant lacking exon 3 is more predominantly expressed in testis [39,43]. Canine retinal transcripts also show exon 3 spliced-in.

The interaction between SPATA7 and the protein complexes that maintain the ciliary gate/barrier regulate both ciliogenesis and protein trafficking within the cilium [30]. In the newly identified distal zone of the connecting cilium (CC), SPATA7 interacts with other photoreceptor-specific proteins, e.g., RPGR, RPGRIP1 and others [30]. SPATA7 is not only necessary for ciliogenesis and photoreceptor development; but in mature photoreceptors, it is required continuously to maintain the integrity of the CC; when eliminated in conditional KO mice, rapid photoreceptor degeneration ensues [40].

In murine photoreceptors, stochastic optical reconstruction microscopy has been used to analyze the connecting cilium, which can be divided in proximal and distal, identifying the latter as a photoreceptor-specific extension of the ciliary transition zone, and highlighting the role of SPATA7 in its maintenance [44]. A recent report using U-ExM showed that SPATA7 is localized surrounding the tubulin axoneme, and confirmed its localization at the distal zone of the connecting cilium in canine photoreceptors [29]. In addition, Eblimit et al. suggested that photoreceptor-specific loss of *Spata7* in can result in altered trafficking of cilium proteins and Rpgrip1 mislocalization in the connecting cilium [41], and that loss of *Spata7* in mouse is sufficient to cause photoreceptor degeneration, suggesting an essential role in both rod and cone function and survival [44].

U-ExM was carried out on photoreceptor cells from one affected dog; furthermore, skin fibroblasts that were preconditioned to undergo ciliogenesis and used as an additional surrogate model system. In photoreceptor, SPATA7 is shown in the ciliary axoneme while PTPN21 is not in the control. SPATA7 signal is absent in the case. The U-ExM imaging of fibroblasts in this study demonstrated that endogenous SPATA7 protein is located near the transition zone above the mother centriole in primary cilia, whereas this signal was absent in cells from an affected dog. The consistent results with two different antibodies and two different cellular tissues strongly suggests that loss of SPATA7 in the affected dogs leads to the mislocalization of key ciliary proteins, similar to that observed in the *Spata7*^*-/-*^ KO mouse [30]. Conversely, PTPN21 was expressed near the base of the primary cilium in fibroblasts from an affected and a control dog in conventional non-expanded immunostaining. Since the Golgi apparatus is distributed near the base of the primary cilium [45], the subcellular localization of PTPN21 observed in fibroblasts aligns with a previous report indicating the localization of PTPN21 in the endosomes and the Golgi apparatus [46].

Wang first identified variants in SPATA7 associated with Leber Congenital Amaurosis (LCA) [36]. The authors observed that LCA variants were centrally positioned in the SPATA7 transcript, while early onset RP variants tended to occur in the last two exons. More than 90% of the reported SPATA7 pathogenic variants in human are truncating in nature (frameshifts and nonsense). However, missense variants and compound heterozygous combinations are reported [38,39,43,47,48]. In addition, a large deletion [49] also has been identified, which shows a high degree of variability in the type of variants reported. Therefore, it appears that several heterogeneous genetic variants in the *SPATA7* gene lead to disease with a similar cluster of pathological features. Observably, the effect of the canine *[SPATA7/PTPN21]DEL* variant is comparable to frameshifts and premature stop codons affecting the terminal part of SPATA7, in a similar way to findings reported by Sengillo and Lee [38].

In conclusion, while the two genes, *SPATA7* and *PTPN21* are expressed in canine retina, especially cone and rod photoreceptors, our studies clearly demonstrate that the deficiency in SPATA7 and not PTPN21 is responsible for the early onset Day Blindness/Retinal Degeneration phenotype present in SP that models the LCA and cone-rod dystrophy phenotype reported in some patients [37,39,50].

## Materials and methods

### Ethical Statement

The research was conducted in full compliance with the Association for Research in Vision and Ophthalmology (ARVO) Resolution on the Use of Animals in Ophthalmic and Vision Research, and approved by the University of Pennsylvania Institutional Animal Care and Use Committee (IACUC - 806301).

### Canine sample pool

Standard poodles manifesting severe or complete day blindness were used in the study. The disease was initially termed achromatopsia as it was clinically similar to a previously identified genetic disorder in other breeds which was referred to as achromatopsia or cone degeneration [51–53]. Initially, seven dogs (SP03, SP04, SP05, SP06, SP07, SP12, SP20, S1 Fig) were examined by referring veterinary ophthalmologists and/or one or more of the authors (GDA, VLD, WAB), and their family information collected. Eight more SPs were later identified and added to the dataset to compare the phenotypes with our genetic findings. These cases, including blood samples, clinical examination records, and, where possible, complete pedigrees, were submitted to a canine DNA testing company (OptiGen LLC, Ithaca, NY) founded by one of the authors (GDA) for archiving and use in gene discovery and mutation identification.

Additionally, we outcrossed an affected dog (SP12) to two non-affected mix-breed females and backcrossed the progeny to SP12; the descendants included non-affected dog SP15 and affected dogs SP16 and SP17. In addition, cases were collected after the mapping and identification of the proposed variant (Table 1, 21 affected dogs in total). Tissue from SP29, an affected pup that died from unrelated medical issues, was subsequently collected and included in the study. For candidate variant genotyping, 63 unaffected SPs were added to the sample pool. Finally, a Goldendoodle (Golden retriever and SP intercross backcrossed to a SP) showing the same phenotype was added to the sample pool after variant identification.

### Pedigree analysis

Described in S3 File

### Clinical and non-invasive studies

Samples obtained from the OptiGen archive came from dogs that had been examined by board certified veterinary ophthalmologists (DACVO) and determined to have severe visual impairment mainly under bright photopic conditions and, in young dogs, absence of any ocular abnormalities based on dilated eye examination using indirect ophthalmoscopy and biomicroscopy. Electroretinography (ERG) was performed on three of the dogs (Table 1, SP09, SP10 and SP18), and all showed absence of cone responses. Focal atrophy of the fovea-like region [54], evident by focal hyperreflectivity, was not recognized or noted in any of these clinical cases. However, one case showed this lesion on the fundus pictures that accompanied the case record while a second one did not.

#### Clinical testing.

The dogs examined in our research facility (Table 1: SP07, SP12, SP15, SP16, SP17, SP20) received a dilated fundus examination, slit-lamp biomicroscopy and fundus photography (Kowa RC2, Genesis or RetCam retinal cameras). Subjective vision testing was done in the examination area under dim and standard room light conditions with obstacles placed at random locations in the room.

#### Electroretinography.

Full-field flash ERGs carried out in our research facility were recorded with an Espion ERG system (Diagnosys, Lowell, MA, USA) under general anesthesia (induction with intravenous propofol; maintenance with isoflurane) using a custom-built Ganzfeld dome fitted with the LED stimuli of a ColorDome stimulator (Diagnosys) as previously described [55,56]. Waveforms were processed with a digital low-pass 50-Hz filter to reduce recording noise if necessary. After 20 min of dark adaptation, rod- and mixed rod-cone-mediated responses (averaged 4 times) to single 4-ms white flash stimuli of increasing intensities (from -3.74 to 0.5 log cd• s m^-2^) were recorded. After 5 min of white light adaptation (1.025 log cd• s m^-2^), cone-mediated signals (averaged 10 times) to a series of single flashes (from 2.74 to 0.5 log cd• s m^-2^) and to 5-Hz (averaged 20 times; from 2.74 to 0.25 log cd• s m^-2^) and 29.4-Hz flicker (averaged 20 times; from 2.74 to 0.25 log cd• s m^-2^) stimuli were recorded. These protocols separately assessed rod- and cone-mediated responses [56].

### Mapping of the causative variant

#### Dogs and single nucleotide variant genotyping.

Blood or buccal swab-derived genomic DNA samples from 84 SPs, 21 affected and 63 unaffected were collected. Of these, 6 cases and 46 controls were used for SNP chip genotyping performed using the CanineHD BeadChip (Illumina, San Diego, CA), see Table 1. For the first batch of 6 cases and 16 controls we used the 170k chip; the version containing 220k evenly distributed SNPs was used for the subsequent genotyping of the remaining 30 dogs, following standard protocols as recommended by the manufacturer. SNP data deposited in Dryad repository [57].

#### Genome-wide association.

We used the GenABEL package for GWAS [58]. Quality control removed markers and individuals with call rates <95%, markers with <5% minor allele frequency (MAF), and markers strongly deviating from Hardy-Weinberg equilibrium. The preliminary MDS plot and ancestry analysis confirmed a stratified population; therefore, the association was completed using a mixed model. The Manhattan plot was analyzed to search for suggestive or associated peaks, prioritizing markers above the Bonferroni correction.

#### Homozygosity mapping.

Homozygosity mapping was carried out with PLINK v.1.9 (“--homozyg” and “--homozyg group” commands) to detect extended intervals of homozygosity with shared alleles [59]. No minimal size threshold for homozygous intervals was specified.

#### Whole genome sequencing.

DNA from the affected SP03 (**Table 1**) was used for whole genome sequencing, and the reads mapped to the dog reference genome canFam4 (UU_Cfam_GSD_1.0, 353,260,936 paired-end reads generated by Casava 1.8). After alignment, the SAM file obtained by Burrows-Wheeler Aligner (BWA) was converted to a BAM file and sorted using SAMtools [60] and after removing PCR duplicates with Picard tools (http://sourceforge.net/projects/picard/) the sorted BAM files were visualized using Integrative Genomics Viewer (IGV) [61]. Fastq deposited in Dryad repository [57].

#### SNV and short indel discovery.

Variant calling was carried out using GATK (version 2.4.9) in the “HaplotypeCaller” mode, with the output set to variant call format (vcf v4.0); the raw calls for all samples and sites were flagged using the standard variant filtration module of GATK (see “best practice” documentation of GATK, version 4) [62]. The prediction of the functional impact of the called variants was done with SnpEff, comparing the data with the canFam4 assembly [63]. Discordant variants also were verified visually using IGV and compared to a database of BAM file our group generated from internal projects and the Dog10K genome project [26,27] to filter out any variant occurring in control dogs.

As additional filtering, candidate variants were converted to the CanFam3.1 reference using the UCSC LiftOver remapping service (https://genome.ucsc.edu/cgi-bin/hgLiftOver, accessed 08/03/2025). Candidate variants in the homozygous interval present in the cases were also filtered against the Dog Biomedical Variant Database Consortium (DBVDC), using the software BCFtools [58], plus additional searching was done in the European Variation Archive variant browser (https://www.ebi.ac.uk/eva/?Variant-Browser, accessed 08/03/2025).

#### Structural variants and mobile elements discovery.

Delly2 was used to detect five types of structural variants in the BAM files: duplications, inversions, insertions, deletions and translocations [64]. BAM files from unrelated dogs of other breeds generated by our group were used as controls. The commands for each of the possible variant type were executed separately and the variants called verified in the called vcf of the internal controls Each of these analyses was carried out focusing on the mapped candidate region which was also visually scanned on integrative genome browser [61]. The variants were then filtered again with internally generated data and the Dog10K genome project [26,27] to check if they occurred in the same or other breeds.

### Variant genotyping

The *SPATA7/PTPN21* deletion boundaries were verified in the cases by re-sequencing targeted PCR products using Sanger sequencing, and data were visualized using 4Peaks (https://nucleobytes.com/4peaks/). PCR primers were designed using PRIMER3 [65], amplified with AmpliTaqGold360Mastermix (Life Technologies, Carlsbad, CA), and products were run on 1.5% agarose gel, 0.5 μg/mL ethidium bromide. All the coordinates are canFam4. Using flanking primers for the *SPATA7/PTPN21* deletion, three primers were designed: a shared reverse primer R (GATCGGGACTCTGGGATTGT, chr8:60,040,599–60,040,618), and two forward primers: F1 (GGCCTATAGAGAGTGGCATGA, chr8: 60,022,391–60,022,410) anneals on one side of the deletion, and F2 (AAGAGAGAGCCCCTGGTTTC, chr8:60,040,187–60,040,206) is within the deletion. F2-R PCR is amplified only in wild-type alleles (431 bp), because the F2 position is missing in cases. The F1-R amplification only happens in mutant alleles (360 bp) – the distance is too long to be amplified in the wild-type allele. The products and the boundaries of the deletion were verified by Sanger sequencing.

### Estimation of the age of the mutation

Described in S3 File.

### RNA transcript analysis and RT-PCR

Described in S3 File.

### Single-cell RNA sequencing analysis of normal canine neuroretina

Neuroretinal punches were obtained from two normal male dogs (age = 40 weeks). The samples were pretreated with 0.25% trypsin for 10 minutes and dissociated into single cells using the Miltenyi Biotec Neural Tissue Dissociation Kit – Postnatal Neurons (Cat. No. 130-094-802, Miltenyi Biotec Inc., Auburn, CA, USA). For each sample, approximately 10,000 cells were loaded onto the 10X Genomics Chromium Next GEM Chip G, and cDNA libraries were prepared using the Chromium Next GEM Single Cell 3’ Reagent Kits v3.1, following the manufacturer’s protocol (10X Genomics, Pleasanton, CA, USA). Sequencing was performed on the Illumina NextSeq 500 platform (Illumina, San Diego, CA, USA).

Sequencing data were processed using the Cell Ranger pipeline (10X Genomics), with alignment to the Canis familiaris reference genome (canFam6). Raw gene expression matrices for each sample (N350 and N354) were imported into Seurat (v5.0.0) using the Read10X and CreateSeuratObject functions, with a minimum threshold of 200 detected features per cell and a requirement of at least 3 cells expressing each gene.

Quality control (QC) metrics were calculated on the merged dataset using the PercentageFeatureSet function. Cells with >5% mitochondrial gene content were excluded to remove low-quality or apoptotic cells. Following initial QC filtering, a total of 10,264 cells were retained. Putative doublets were identified and removed using DoubletFinder (v2.0.6), with parameter optimization performed using paramSweep and find.pK. After doublet removal, 9,853 high-confidence singlet cells remained for downstream analysis.

Each dataset was normalized using the SCTransform function. The top 3,000 most variable features were selected using SelectIntegrationFeatures, and datasets were integrated using FindIntegrationAnchors and IntegrateData (SCT workflow). Dimensionality reduction was performed using principal component analysis (PCA) followed by Uniform Manifold Approximation and Projection (UMAP), based on the top 30 principal components. The final integrated dataset included 9,853 high-quality singlet cells.

Data scaling was performed with ScaleData, and dimensionality reduction was further refined using RunPCA and RunUMAP. Graph-based clustering was conducted using FindNeighbors and FindClusters (resolution = 0.5), and cluster identities were assigned based on the expression of established retinal marker genes (shown in **Fig 3**) Marker gene expression and cluster identities were visualized using DotPlot, FeaturePlot, and VlnPlot functions. Specific expression patterns of PTPN21 and SPATA7 were visualized using violin plots.

### Immunocytochemistry of canine retina and skin fibroblasts

The dissociated fibroblasts from canine skin biopsies were cultured, and ciliogenesis was induced by serum deprivation. The U-ExM procedure followed an optimized protocol for observing the primary cilium in both fibroblasts and retinal samples [29,66]. Methods used for the analysis of the affected and wild-type canine fibroblast cilia are reported in detail in S3 File.

#### *PTPN21* canine transcript structure analysis.

The *PTPN21* canine transcript was analyzed to verify the impact of the 3’ deletion. In detail, the occurrence in retina of a split *PTPN21* transcript, with the 5’ transcript being unaffected by the deletion is detailed in S1 File.

#### *Ptpn21* knockout.

Mice from a *Ptpn21* KO colony where a fragment of *Ptpn21* was deleted were used for morphologic evaluation of the retina. These mice show undetectable *Ptpn21*^-/-^ mRNA in early progenitor LSK (lineage-negative Sca-1 + cKit+) cells enriched for hematopoetic stem cells, but show no overt abnormalities in the first 12 months of life [31]. Eyes from *Ptpn21 + /+* and homozygous KO Ptpn21^-/-^ mice, each pair 1.6 and 5.3 months of age, were collected after euthanasia, fixed in modified Davidson’s solution, trimmed, paraffin embedded, sectioned at 6 µm thickness and stained with H&E for microscopic examination or processed for immunohistochemistry with antibodies against rod opsin and S- and M-cone opsins [55,67] Slides were examined by transmitted light or epifluorescence illumination with a Zeiss Axioplan microscope (Carl Zeiss Meditec, Thornwood, NY).

## Supporting information

S1 Fig Subset pedigrees of the SNP genotyped cases, their close relatives and initial homozygosity mapping. (**A**) Red filled symbol are dogs clinically affected. Males are indicated with squares; females are indicated with circles; half-filled symbols are putative heterozygotes. Family history indicated that the parents and siblings were unaffected suggesting recessive inheritance (not all family members are included). All the cases were genotyped on SNP chip along with 46 controls. (**B**) Homozygosity mapping of the six cases, two available unaffected siblings and two additional unrelated controls. The homozygous regions shared by all the cases and exclusive to them are marked in red. Observe the low number of small, shared intervals. With an asterisk, the CFA8 interval highlighted by GWAS is shown, overlapping with the shared CFA8 homozygous region.
(TIF)

S2 Fig Family trees of all the cases whose ancestry could be reconstructed. Black filled symbol are dogs clinically affected with the del/del genetic variant. Males are indicated with squares, females are indicated with circles; half-filled symbols are genotyped or putative heterozygotes. The blue arrow indicates the dog which was whole-genome sequenced. The red arrow shows the identified putative common ancestor, born in 1923. Curved lines indicate the same dog that is shown in more than one position in the pedigree.
(TIF)

S3 Fig (A) Detailed view of the Manhattan plot showing CFA8. In addition to the Manhattan plot, on top left the QQ-plot shows the observed quantiles (y axis) log p-values, versus the expected ones on the x axis. The distinct skewing of a marker toward the upper side confirms the association with the affected condition compared with the expected values in case of mere chance. (**B**) Detail of the associated region. Aff: the six genotyped cases. Con: the 46 controls. The shared homozygous region (Canfam3:1; chr8:59,290,908–60,234,084) is shown highlighted by a black box. The seven most associated SNPs are shown as red arrowheads. Note that the best associated SNPs from the GWAS analysis fall outside the critical candidate region. (**C**) Genotyped variant. Top: Position of the break points and primer placement are shown (see Materials and Methods). Bottom: results of the PCR amplification for affected (“A”), Carriers (“Ca”) and Wild Type (“WT”) Standard poodles. Ladder indicated as “Mk”.
(TIF)

S4 Fig (A) paired-end RNA-seq from skin fibroblasts obtained from normal Standard poodle (top, wild-type), a carrier for the genetic variant for *SPATA7* (Heterozygous), a homozygous mutant, and (bottom) the genomic interval of the sequenced case showing the large deletion of the 3’ parts of *SPATA7* and *PTPN21.* Note the presence of the last two exons in the control and carrier, while in the mutant there are no reads that align on the annotated last exons of *SPATA7* and *PTPN21* – the read pairs occasionally go across one side to the other of the gap. (**B**) detail of the fibroblast RNA-seq in a WT and mutant *SPATA7* exon 9. Note the splicing of exon 9 (exons 8 and 10) in the WT and the lack exon 10 splicing in the mutant. The ins[g.60,018,954–60,018,990] is highlighted in purple and the frameshift and premature stop indicated. (**C**) Resulting predicted truncated SPATA7 protein (Asp361Glu Substitution and part predicted to be lost due to the following premature stop codon marked with a red line). The variant is compared with selected causative *SPATA7* mutations in humans (see Discussion for mutation details and references), position marked with grey arrowheads (The variant reported in Mayer et al. which deletes ORF of *SPATA7*, is marked with the grey bar) [49].
(TIF)

S5 Fig Localization of endogenous SPATA7 and PTPN21 protein in ciliated canine skin fibroblasts. Subcellular localization of SPATA7 (A, green) and PTPN21 (B, green) in *SPATA7*^*+/+*^ and *SPATA7*^*-/-*^ canine-derived skin fibroblasts induced for ciliogenesis by 48 h of serum starvation. Both conventional immunocytochemistry (upper panels) and U-ExM (lower panels) images are shown. (**A**) In non-expanded cells, endogenous SPATA7 signals were not detected around the primary cilium in either *SPATA7*^*+/+*^ or *SPATA7*^*-/-*^ cilia. U-ExM revealed a distinct SPATA7 signal surrounding the transition zone of the primary cilium in *SPATA7*^*+/+*^ fibroblasts (white arrowhead), which was absent in *SPATA7*^*-/-*^ cells (black arrowhead). Insets show magnified views of the basal region of the primary cilium. (**B**) Endogenous PTPN21 was detected near the base of the primary cilium in non-expanded samples (yellow arrowheads) but was not detectable in U-ExM samples. No obvious difference in PTPN21 localization was observed between *SPATA7*^*+/+*^ and *SPATA7*^*-/-*^ fibroblasts. Scale bars: 1 μm (no-expansion) and 5 μm (U-ExM), without correction for expansion factor. BB, basal body; DC, daughter centriole.
(TIF)

S1 File Analysis of the canine retinal *PTPN21* transcript and comparison with other tissues. The analysis shows that in retina *PTPN21* is split in two transcripts at exon 12; only one of these transcripts (3’ direction) is affected by the deletion associated with retinal degeneration in Standard poodle. PTPN21 contains two domains (PTP domain, a FERM), which are encoded by one of the two transcripts each. This suggests that the variant is not as impactful on PTPN21 as it is in SPATA7, which has no alternate transcripts in retina not affected by the deletion [69,70].
(DOCX)

S2 File Variant impact on the SPATA7 sequence. (DOCX)

S3 File Description of methods not reported in the main body of text. References are also indicated in the main article [29,61,65,66,71–77].
(DOCX)
