# Supplementary figures and images for: Two genes, one culprit - a functional candidate validation of a SPATA7 deletion in dogs with day blindness/retinal degeneration
Source: PLoS Genet. 2025 Dec 1;21(12):e1011961. doi: 10.1371/journal.pgen.1011961 (PMC12680346; doi:10.1371/journal.pgen.1011961)

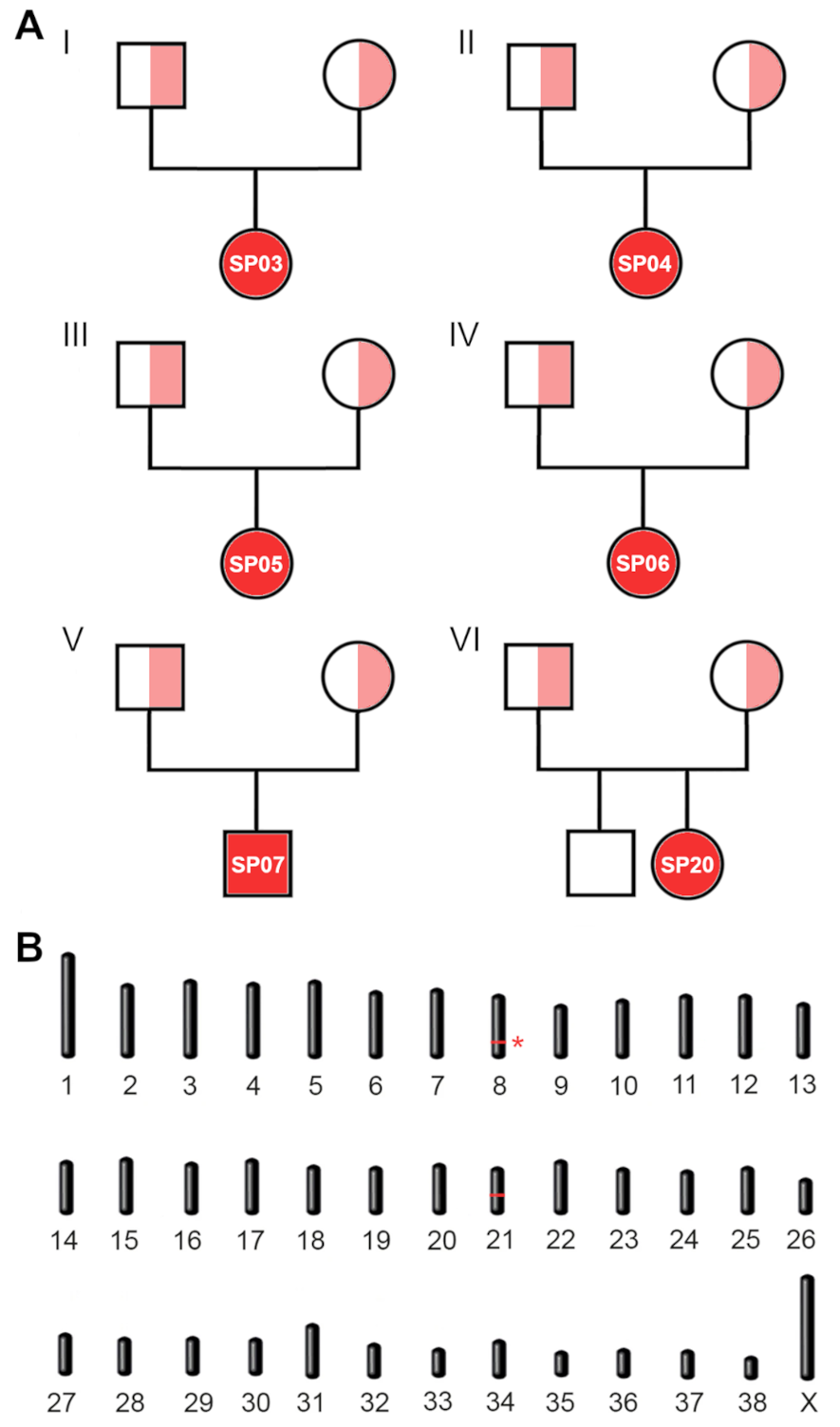

Supplement: S1 Fig — (A) Red filled symbol are dogs clinically affected. Males are indicated with squares; females are indicated with circles; half-filled symbols are putative heterozygotes. Family history indicated that the parents and siblings were unaffected suggesting recessive inheritance (not all family members are included). All the cases were genotyped on SNP chip along with 46 controls. (B) Homozygosity mapping of the six cases, two available unaffected siblings and two additional unrelated controls. The homozygous regions shared by all the cases and exclusive to them are marked in red. Observe the low number of small, shared intervals. With an asterisk, the CFA8 interval highlighted by GWAS is shown, overlapping with the shared CFA8 homozygous region. (TIF) [file pgen.1011961.s001.tif]

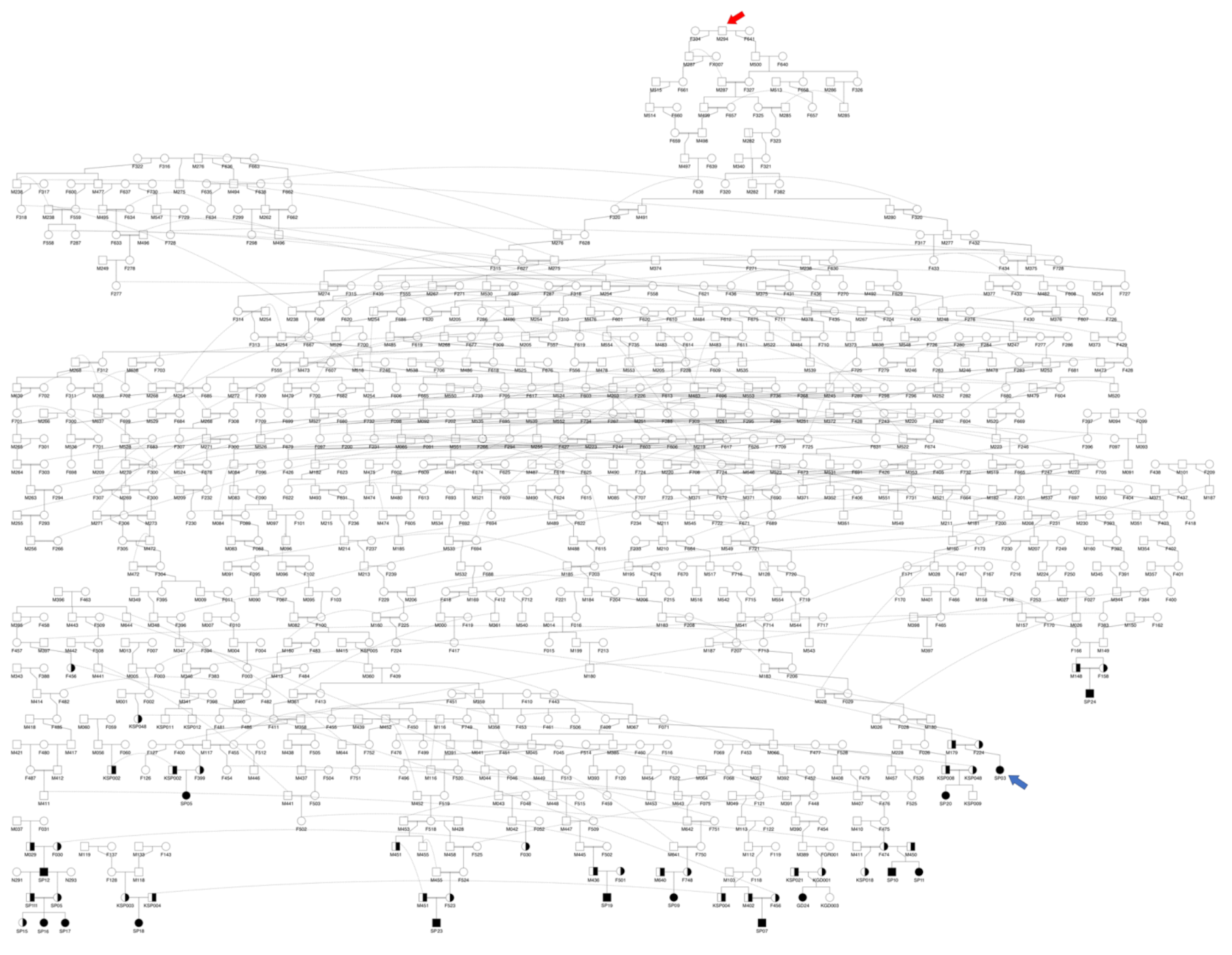

Supplement: S2 Fig — Black filled symbol are dogs clinically affected with the del/del genetic variant. Males are indicated with squares, females are indicated with circles; half-filled symbols are genotyped or putative heterozygotes. The blue arrow indicates the dog which was whole-genome sequenced. The red arrow shows the identified putative common ancestor, born in 1923. Curved lines indicate the same dog that is shown in more than one position in the pedigree. (TIF) [file pgen.1011961.s002.tif]

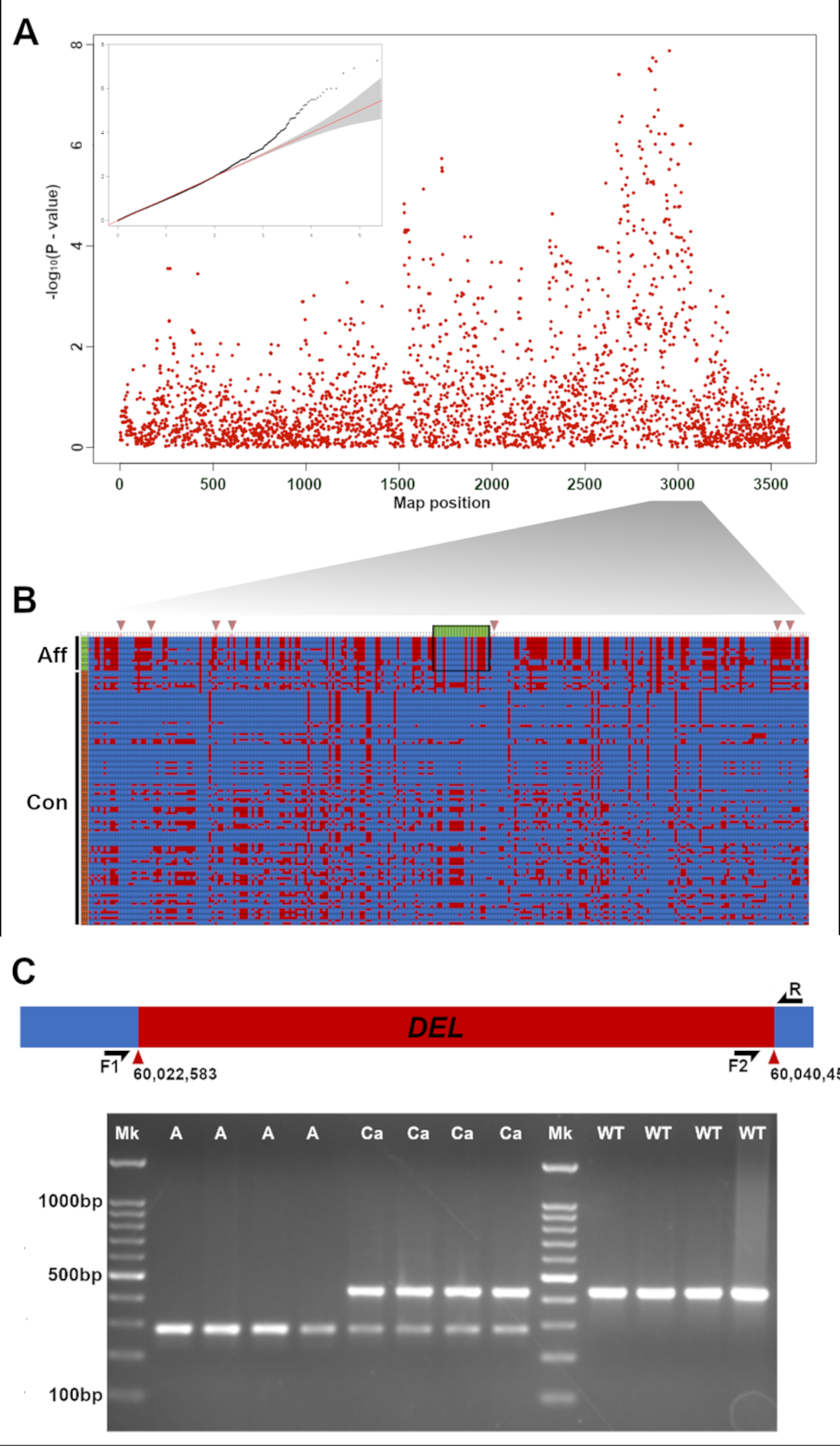

Supplement: S3 Fig — In addition to the Manhattan plot, on top left the QQ-plot shows the observed quantiles (y axis) log p-values, versus the expected ones on the x axis. The distinct skewing of a marker toward the upper side confirms the association with the affected condition compared with the expected values in case of mere chance. (B) Detail of the associated region. Aff: the six genotyped cases. Con: the 46 controls. The shared homozygous region (Canfam3:1; chr8:59,290,908–60,234,084) is shown highlighted by a black box. The seven most associated SNPs are shown as red arrowheads. Note that the best associated SNPs from the GWAS analysis fall outside the critical candidate region. (C) Genotyped variant. Top: Position of the break points and primer placement are shown (see Materials and Methods). Bottom: results of the PCR amplification for affected (“A”), Carriers (“Ca”) and Wild Type (“WT”) Standard poodles. Ladder indicated as “Mk”. (TIF) [file pgen.1011961.s003.tif]

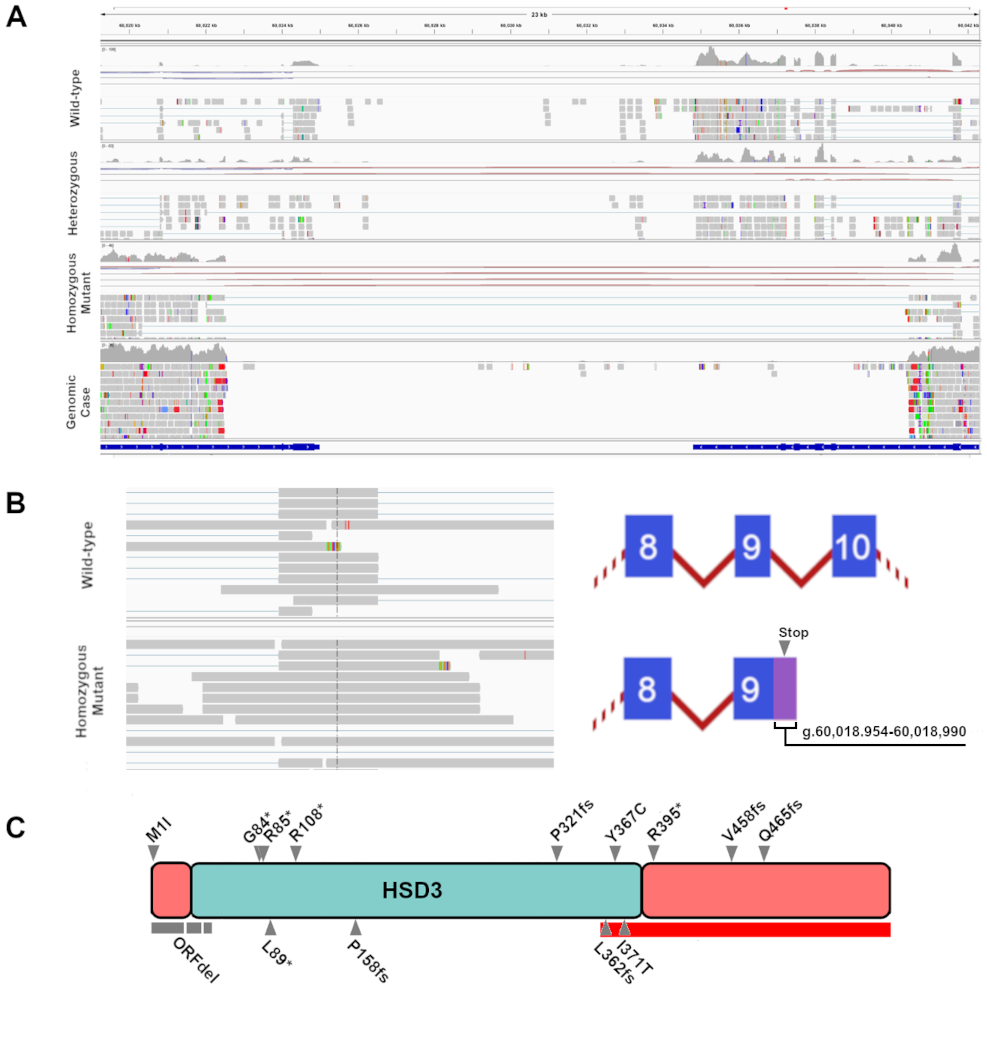

Supplement: S4 Fig — Note the presence of the last two exons in the control and carrier, while in the mutant there are no reads that align on the annotated last exons of SPATA7 and PTPN21 – the read pairs occasionally go across one side to the other of the gap. (B) detail of the fibroblast RNA-seq in a WT and mutant SPATA7 exon 9. Note the splicing of exon 9 (exons 8 and 10) in the WT and the lack exon 10 splicing in the mutant. The ins[g.60,018,954–60,018,990] is highlighted in purple and the frameshift and premature stop indicated. (C) Resulting predicted truncated SPATA7 protein (Asp361Glu Substitution and part predicted to be lost due to the following premature stop codon marked with a red line). The variant is compared with selected causative SPATA7 mutations in humans (see Discussion for mutation details and references), position marked with grey arrowheads (The variant reported in Mayer et al. which deletes ORF of SPATA7, is marked with the grey bar) [49]. (TIF) [file pgen.1011961.s004.tif]

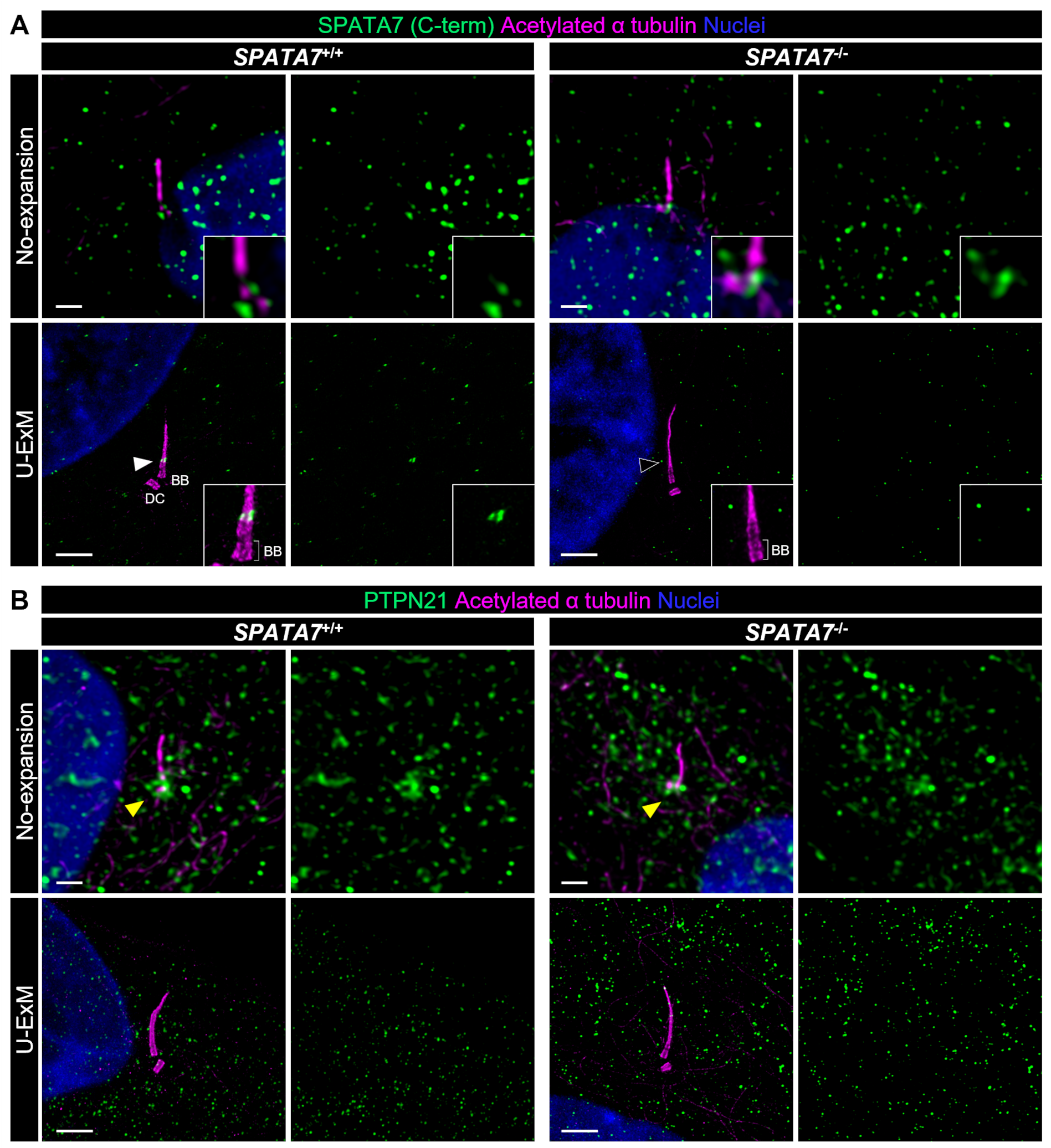

Supplement: S5 Fig — Subcellular localization of SPATA7 (A, green) and PTPN21 (B, green) in SPATA7+/+ and SPATA7-/- canine-derived skin fibroblasts induced for ciliogenesis by 48 h of serum starvation. Both conventional immunocytochemistry (upper panels) and U-ExM (lower panels) images are shown. (A) In non-expanded cells, endogenous SPATA7 signals were not detected around the primary cilium in either SPATA7+/+ or SPATA7-/- cilia. U-ExM revealed a distinct SPATA7 signal surrounding the transition zone of the primary cilium in SPATA7+/+ fibroblasts (white arrowhead), which was absent in SPATA7-/- cells (black arrowhead). Insets show magnified views of the basal region of the primary cilium. (B) Endogenous PTPN21 was detected near the base of the primary cilium in non-expanded samples (yellow arrowheads) but was not detectable in U-ExM samples. No obvious difference in PTPN21 localization was observed between SPATA7+/+ and SPATA7-/- fibroblasts. Scale bars: 1 μm (no-expansion) and 5 μm (U-ExM), without correction for expansion factor. BB, basal body; DC, daughter centriole. (TIF) [file pgen.1011961.s005.tif]
