# Supplementary material for: Two genes, one culprit - a functional candidate validation of a SPATA7 deletion in dogs with day blindness/retinal degeneration
Source: PLoS Genet. 2025 Dec 1;21(12):e1011961. doi: 10.1371/journal.pgen.1011961 (PMC12680346; doi:10.1371/journal.pgen.1011961)
Supplement: S1 File — The analysis shows that in retina PTPN21 is split in two transcripts at exon 12; only one of these transcripts (3’ direction) is affected by the deletion associated with retinal degeneration in Standard poodle. PTPN21 contains two domains (PTP domain, a FERM), which are encoded by one of the two transcripts each. This suggests that the variant is not as impactful on PTPN21 as it is in SPATA7, which has no alternate transcripts in retina not affected by the deletion [69,70]. (DOCX) [file pgen.1011961.s006.docx]

**S1 File.** Analysis of the canine retinal *PTPN21* transcript and comparison with other tissues. The analysis shows that in retina *PTPN21* is split into two transcripts at Exon 12; only one of these transcripts (3’ direction) is affected by the deletion associated with retinal degeneration. PTPN21 contains two domains (PTP domain, a FERM), which are situated in one of the two transcripts each. This suggests that the variant is not as impactful on *PTPN21* as it is in *SPATA7*, which has no alternate transcripts in retina not affected by the deletion.

**PTPN21 role and deleted isoform**

***Background***

The protein tyrosine phosphatase non-receptor type 21 (*PTPN21*) gene consist of 19 total exons with 2 reported predicted isoforms: canFam4 (henceforth) XM_038545501.1 and XM_038545502.1, which differ only in their 5’ UTR. Both contain 18 coding exons, and both are predicted to encode a 1,170-amino acid protein (XP_038401429.1). Gene structure is highly conserved between species, including a long 1,421-bp 12^th^ coding exon, (**Figure S1-1A, B)**. The variant we report has coding exons 15 through 18 removed; these are predicted to encode amino acids 954 through 1170 and includes the substrate binding site of the PTP domain (amino acids 1104 through 1110) (**Figure S1-1C**).

***Results***

To predict the impact of the *SPATA7* and *PTPN21* gene deletions, retinal RNA-seq data published by our group [69] from dogs unaffected by the *[SPATA7/PTPN21]DEL* variant were examined, and a break in coverage in the middle of the long 12^th^ exon in the retinal *PTPN21* transcript was noted (**Figure S1-2)**. To characterize this pattern, determine the normal *PTPN21* expression in retina and assess how the variant affects expression, overlapping primer pairs (**Table S1-5**) spanning the coding sequence of canine *PTPN21* were designed and used to amplify RNA extracted from the retinas of four different normal control dogs and RNA extracted from the leukocytes of one normal control dog. Because the predicted canine transcripts have identical coding regions, we expected to amplify one retinal isoform of *PTPN21* and no additional splice variants. It was possible to amplify the *PTPN21* transcript from exon 1 to the beginning of exon 12, and from the end of exon 12 to exon 18, and these regions produced products of the expected size and sequence (**Figure S1-3A)**, but all attempts to amplify through the middle of exon 12 produced inconsistent, nonspecific results (**Figure S1-3B**), despite multiple modifications to the PCR conditions to account for the high GC content of this region. However, it was possible to amplify this region of exon 12 from genomic DNA extracted from a blood sample of a wild-type dog (**Figure S1-3C**), indicating that the primers and thermocycling conditions were sufficient for the amplification of this region. These data suggest that the *PTPN21* transcript could be expressed as two separate transcripts in the retina. This could explain the poor coverage of exon 12 displayed in the RNA-seq data (**Figure S1-2)**, as this region of low-coverage in the RNA-seq data corresponded to the portion of exon 12 that could not be amplified from cDNA.

Previous studies have reported results consistent with *PTPN21* having the potential to be expressed as two transcripts. Tokuchi et al. [70] examined *PTPN21* expression in murine testes and identified two transcript variants, the relative expression of which differed among cell types within the testes. The longer, 5.7-kb transcript corresponds in size to the previously reported full-length *PTPN21* transcript and encodes an ezrin-like domain as well as a catalytic (PTP) domain. The shorter, 2.9-kb transcript contains only the PTP catalytic domain, supporting the idea that *PTPN21* can be expressed as one transcript or two [70].

To determine how the deletion of the last four exons of the gene would affect expression of the *PTPN21* transcript, we amplified two regions of *PTPN21* using probes spanning exons 8 and 9 (which are unaffected by the variant) and probes spanning exons 14 and 15 (which are expected to be deleted in the variant) in cDNA derived from the fibroblasts of one dog with the variant and an unaffected control (**Fig S1-4**). As expected, exons 14 and 15 of the transcript were not detected in the affected dog. In addition, expression of exons 8 and 9 was equivalent to that of the unaffected dog, supporting the hypothesis that the *PTPN21* transcript can be expressed as two separate transcripts. From these results, it can be observed that while the variant removes the active site of the PTP domain, a transcript containing a FERM domain is expressed, showing that the deletion does not affect expression of the 5’ region of *PTPN21*.

**
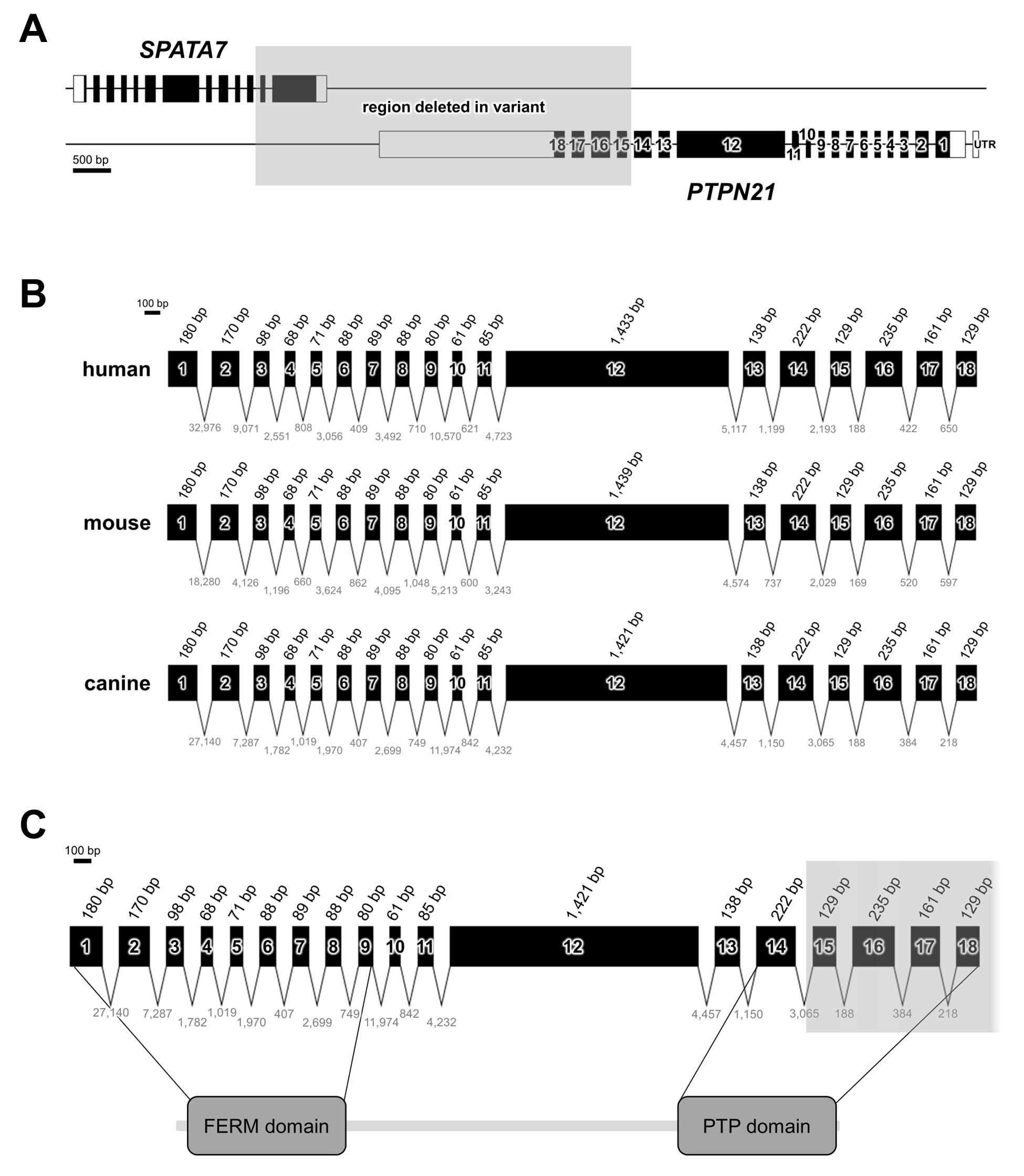
**

**Figure S1-1. Gene structure of canine *PTPN21* based on the canFam4 genomic assembly** (**A**) Schematic of a portion of canine chromosome encoding *SPATA7* and *PTPN21* (on the reverse strand) with the sequence deleted in the variant indicated (*gray box*); scale: 500 bp. (**B**) Schematic of the human (NM_007039.4), mouse (NM_001146199.1), and canine (XM_038545501.1) *PTPN21* coding sequences showing similarities in structures among species; scale: 100 bases. (**C**) Schematic of the canine *PTPN21* gene structure and the corresponding protein domains with the sequence deleted in the variant indicated (*gray box*); scale: 100 bases. Schematics of the transcript were made with the Exon-Intron Graphic Maker tool (http://wormweb.org/exonintron). Exon numbers were assigned with exon 1 as the first coding exon; introns and inter-gene regions are not drawn to scale.

**
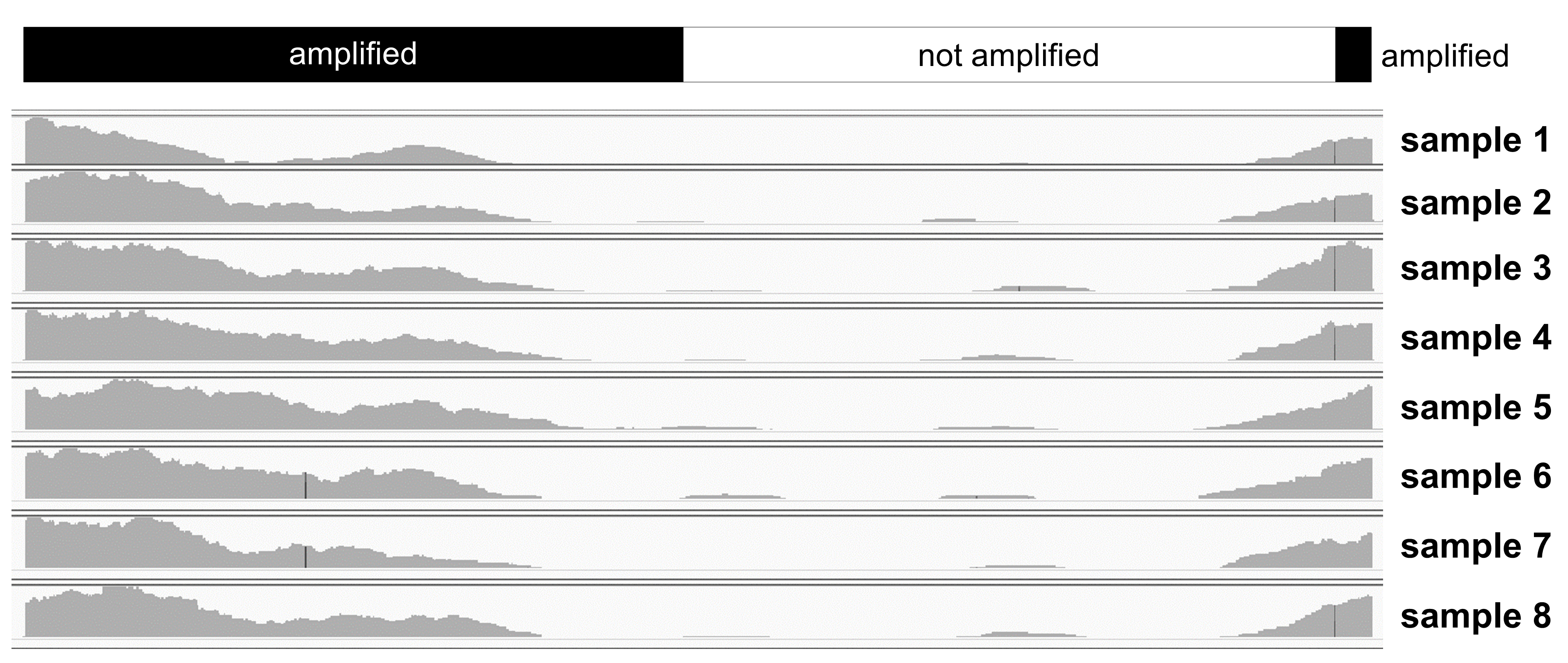
**

**Figure S1-2. Comparison of read coverage in *PTPN21* exon 12.** RNA-seq data from 8 canine wild type retinas was analyzed in the Integrative Genomics Viewer (IGV) and showed poor read coverage for the middle of *PTPN21* exon 12. For reference, a schematic (*above*) showing the approximate locations of the regions of exon 12 that were amplified (*black bars*) and the region that could not be successfully amplified from retinal cDNA (*white bar*) is included.


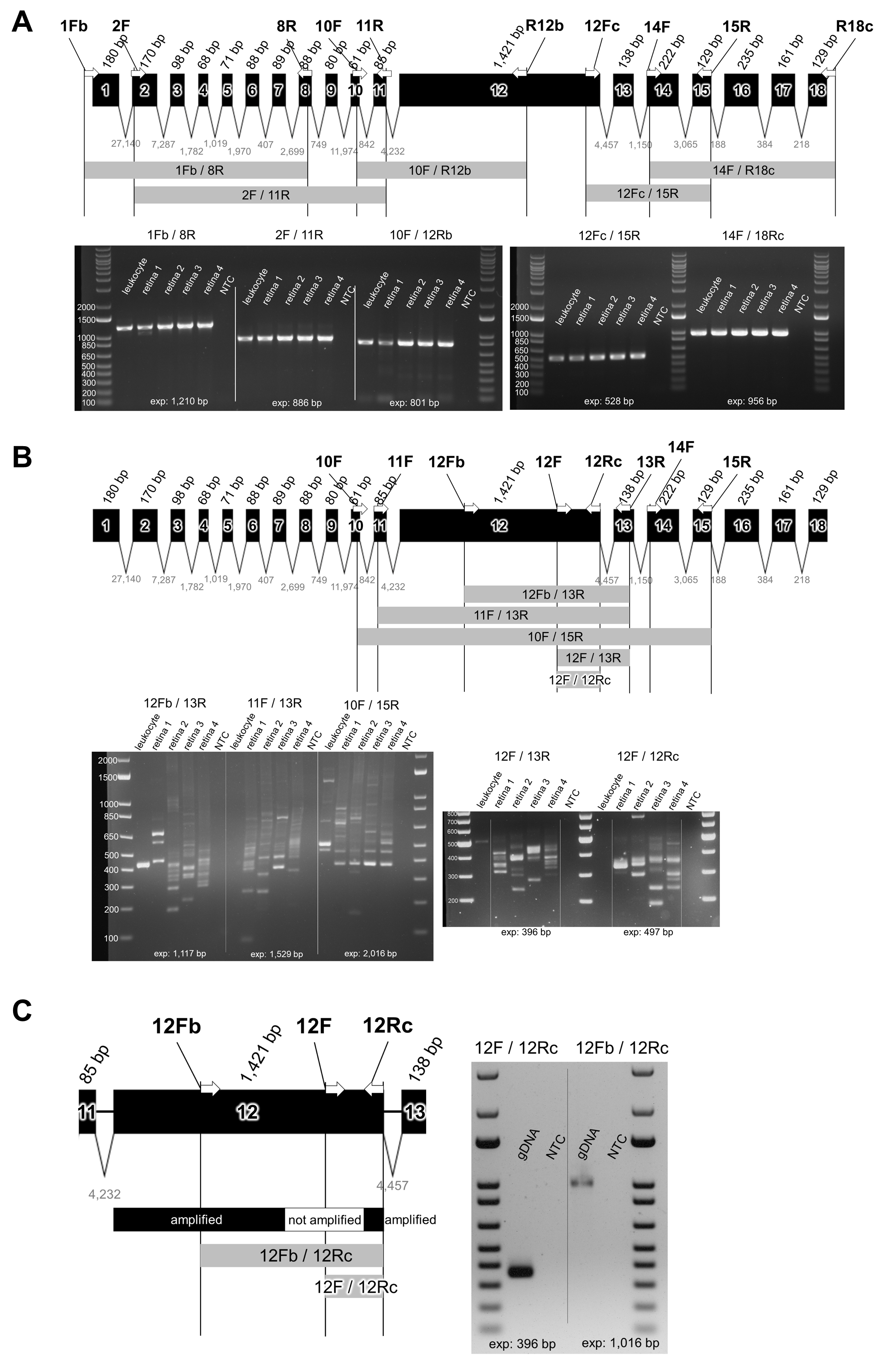


**Figure S1-3. Amplification of retinal cDNA from four different wild-type dogs using primers to the *PTPN21* transcript.** Leukocyte-derived cDNA previously used to test the primers was used as a positive control. (**A**) Two sections of the *PTPN21* coding region from exon 1 to the beginning of exon 12 and from the end of exon 12 to exon 18 were successfully amplified from cDNA; schematic showing the approximate location of the primers on the canine *PTPN21* transcript (*above*) and PCR products separated by agarose gel electrophoresis results (*below*); specificity of amplified products were confirmed by Sanger sequencing. (**B**) Amplification of the middle region of *PTPN21* exon 12 from cDNA was unsuccessful, and the products produced were inconsistent among samples and appeared to be non-specific; schematic showing the approximate location of the primers on the canine *PTPN21* transcript (*above*) and PCR products separated by agarose gel electrophoresis results (*below*). Results shown are representative of multiple thermocycling conditions. (**C**) Successful amplification of the middle of *PTPN21* exon 12 from genomic DNA; schematic showing the approximate location of the primers on the canine *PTPN21* transcript (*left*) with a bar (*below left*) showing the regions of exon 12 that were amplified from cDNA (*black*) and the region that could not be amplified (*white*), and PCR products separated by agarose gel electrophoresis results (*right*). Schematics of the transcript were made with the Exon-Intron Graphic Maker tool (http://wormweb.org/exonintron). Exon numbers were assigned with exon 1 as the first coding exon; introns and inter-gene region are not drawn to scale; NTC = no template control.


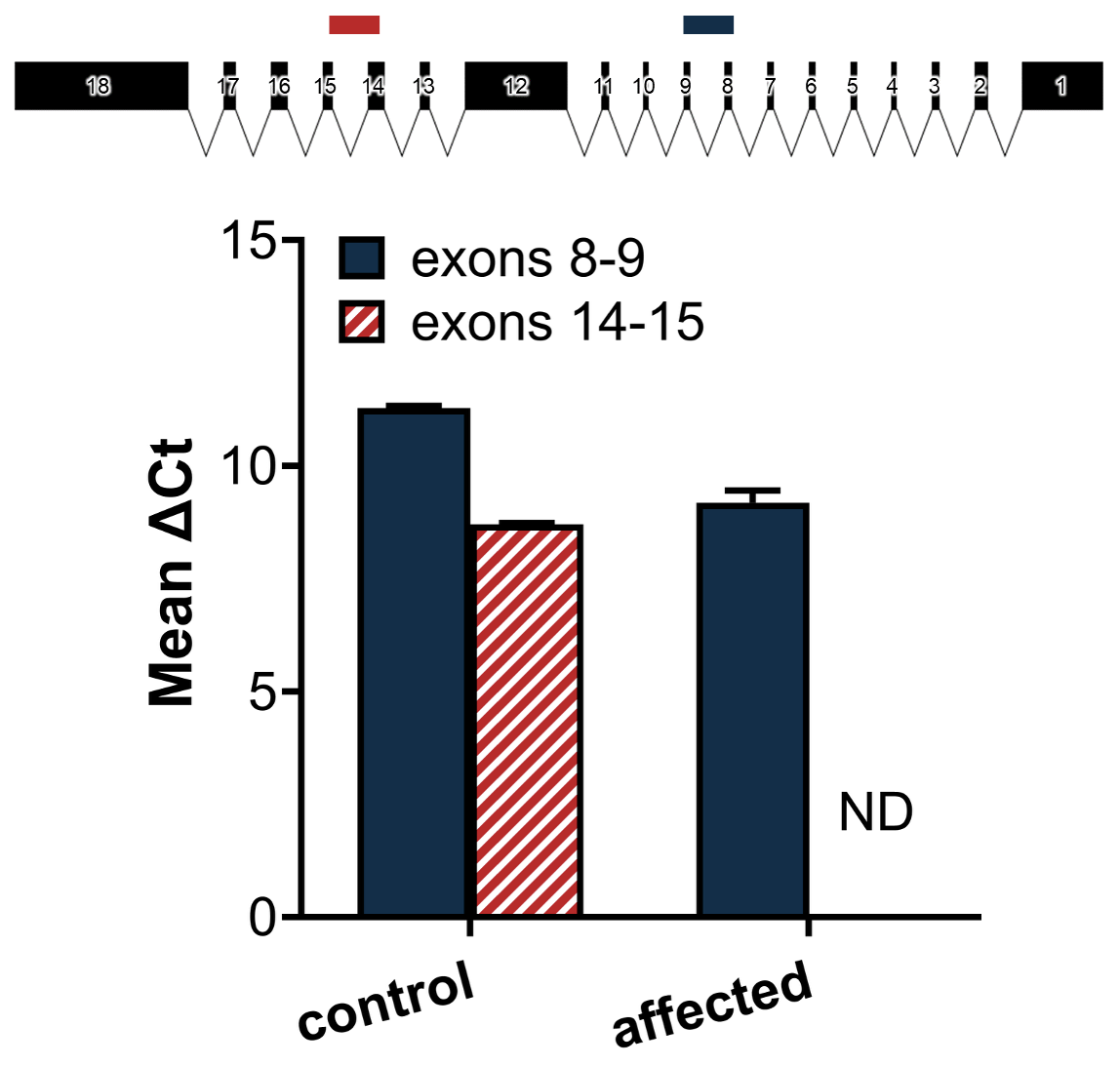


**Fig. S1-4.** Expression of two parts of *PTPN21* in fibroblast cDNA from a control and an affected dog. Schematic of the *PTPN21* transcript, which is encoded on the reverse strand, showing the locations of probes used for the TaqMan assay (top). Mean ΔCt values (± standard deviation, normalized to GAPDH) of a TaqMan assay using probes spanning exons in a region of the PTPN21 transcript unaffected by the variant (exons 8-9) and in a region affected by the variant (exons 14-15). The affected region was not detected (ND) in fibroblast cDNA from the affected dog.

**Table S1-5**. Sequences of primers used to amplify *PTPN21* transcripts in cDNA synthesized from canine retina. Exon numbers were assigned with exon 1 as the first coding exon.

| Primer | Sequence (5’ to 3’) | Exon location (direction) |
| --- | --- | --- |
| 1Fb | CCAGGTCCTGGTGATACCTTC | 5’ UTR (forward) |
| 2F | CAGTCTCTGGTACCACAACAAGC | 2 (forward) |
| 8R | ATGCAAAGAAGGACTTGTTGTGG | 8 (reverse) |
| 10F | TCGTCTTCAAGAGTGTCCCTG | 10 (forward) |
| 11F | AGAACCATACACTTCCTCCCAAG | 11 (forward) |
| 11R | TTGGGAGGAAGTGTATGGTTCTG | 11 (reverse) |
| 12Fb | GTCTACAGCCAGCCAGAGATTC | 12 (forward) |
| R12b | GTTGCTGCTGCTTATGTACAGG | 12 (reverse) |
| 12F | CCACAAGAAGTCTCTGTCTGACG | 12 (forward) |
| 12Fc | GGAAAGAAGAACGTCGTGGAAGG | 12 (forward) |
| 12Rc | CCTTCCACGACGTTCTTCTTTCC | 12 (reverse) |
| 13R | GCAGTCGAGAGAGGGAGAGTC | 12 (reverse) |
| 14F | AGCGGTTAGAACAGGGAATGG | 14 (forward) |
| 15R | CTCCTCTGCAGTCACCATTGC | 15 (reverse) |
| R18c | TCTCGGGTATCGGTGAGGAAG | 3’ UTR (reverse) |

**References**

69. Sudharsan R, Beiting DP, Aguirre GD, Beltran WA. Involvement of Innate Immune System in Late Stages of Inherited Photoreceptor Degeneration. Sci Rep. 2017;7(1):17897. Epub 2017/12/22. doi: 10.1038/s41598-017-18236-7. PubMed PMID: 29263354; PubMed Central PMCID: PMCPMC5738376.

70. Tokuchi H, Higashitsuji H, Nishiyama H, Nonoguchi K, Nagao T, Xue JH, et al. Expression of protein tyrosine phosphatase PTP-RL10 and its isoform in the mouse testis. Int J Urol. 1999;6(11):572-7. Epub 1999/12/10. doi: 10.1046/j.1442-2042.1999.611108.x. PubMed PMID: 10585123.
