# Supplementary material for: Two genes, one culprit - a functional candidate validation of a SPATA7 deletion in dogs with day blindness/retinal degeneration
Source: PLoS Genet. 2025 Dec 1;21(12):e1011961. doi: 10.1371/journal.pgen.1011961 (PMC12680346; doi:10.1371/journal.pgen.1011961)
Supplement: S2 File — (DOCX) [file pgen.1011961.s007.docx]

**S2 File. - Variant impact on SPATA7**

***A1. Re-annotated canine transcript (NOTE: missing exons 11 and 12 as per genomic annotation (namely,*** CFA8:NC_049229.1:g.60,022,583_60,040,453del)***, - see the actual predicted transcript in A2).***

Note: deleted exons fragments are in red and underscored.

>SPATA7_predicted_retinal_canine_transcript

CCTTAGCAACCGGCCTGGCGACGGTGTCGTGCGGCTGCCGGCACGCGCCGCCCCGTGTGCGCCAGTCCTCTCCGGCGGGGGCTGGCCCGGCCCCGGGAAGGTCCGAAGGGGGCCCGGGGGTCCTCACGGCGGCGGCTGCAAGAGGATCTAAGC**ATG**GACGGCAGCCGGAGAGTCAGAGCAACCTCTGTCCTTCCCAGATATGGTACACCATGCCTGTTTAAAGGACACTTGAGCACCAAAAGTAATGCTTTTTGCACTGACTCCTCCTCTCTCAGACTAAGTACTCTCCACCTGGTCAAGAATCACATGGCTGTTCACTATAATAAAATCCTTTCAGCCAAAGCTGCAGTAGACTGCTCGGTTCCATTAAGCATAAGTGCCAGCATCAAATATGCAGACCAACAACGAAGAGAGAAACTTAAAAAGGAATTAGCACGATGTGAAAGGGAGTTGAAATTAAATAAAACAGCAATGCAGGCCAATTCTAAAAATAATTCCAAGTCATTATTTAACACTCTACAAAAGCCCTCAGGAGAACCACAAGATGAAGATGTGTTAATCAGAGAAGTGAATGGATTTCCATCCTTTACAAGGTCACCAGTAACTTCTTCAGAGAGACTACACCTAAATATACCTAAATCTGATAAAGTCCTCACAAATGGTACTGAGAAGAACTCCAGTCCCTGTCTGTTCAACGTGGATTACACCACCTCTGGGCCCAGGAGACCATGCTCTGGAACATCACATGGCCGAGGGCCCAAGAGCACGTTCCCAAATACGCATCGCTTTCAGTTAGTTATTTCAAAAGCACCCAGTGGGGACCTTTTGGATAAACATTCTGAACTCTTTTCCAACAGACATTTGCCATTCACTCCACGCACTTTAAAAACAGAAGCAAAGTCTTTCCTGTCACATTATCGATATTACACACCTGCCAGAAGAAAAAAGGATTTTACAGATCAGCGGATGGAAGCTGAAACCCAGACTGAATTAAGCAGCTTTAAATCTGATTTTGAGACAGTTGACACCAAGAACTTCACAGATTCAGAAGAGAACATAAAGCAGGCATCTAACTGTTTGACAGATGATACCAAAGGAAAAATAAATCCTTTACCCCTACAAGGGCATGACTTACCATGGGATGAGATCAAAGATGGCACTCTTCAGTGCTCCTCACCAAGGGCAGTATGTCAGTATTCCCTGCAGCTTCCTCCAGAGAGAAAAATCTACTCTGATGAAGAAGAACTGTTGTATCTGAGTTTCATTGAAGATGTAACAGATGAAATTTTGAAACTTGGCTTATTTTCAAACAGGTTTTTAGAACGACTGTTTGAGCGACATATAAAACAAAATAAACATCATTTGGAGGAGGGGAAAATGCGCCACCTGCTGCATATCCTGAAGGTGGACTTGGGCTGCACATCCAGAGAAAACTCAGTAAAGCTGGATGATCTTGATATGCTGGATTTACATGATTTTGAACAGGCTGAGAATTCAAAAGAAAATGAATTTAGAAATAAACACGACACCACAATTCAACAGGAACGTCAAGAATACCAAAAAGCTTTGGATATGTTATTGTCTATACCAAAGGATGAGAATGAGAGACTCTCTTCACCAAATGAATTT

***A2. Mutant transcript (RNA-seq fibroblast, case)***RNA-seq predicted mutants highlighted the ins[g.60,018,954-60,018,990], stop codon in red and underscored).

>Mut_SPATA7_Canine_RNAseq

CCTTAGCAACCGGCCTGGCGACGGTGTCGTGCGGCTGCCGGCACGCGCCGCCCCGTGTGCGCCAGTCCTCTCCGGCGGGGGCTGGCCCGGCCCCGGGAAGGTCCGAAGGGGGCCCGGGGGTCCTCACGGCGGCGGCTGCAAGAGGATCTAAGC**ATG**GACGGCAGCCGGAGAGTCAGAGCAACCTCTGTCCTTCCCAGATATGGTACACCATGCCTGTTTAAAGGACACTTGAGCACCAAAAGTAATGCTTTTTGCACTGACTCCTCCTCTCTCAGACTAAGTACTCTCCACCTGGTCAAGAATCACATGGCTGTTCACTATAATAAAATCCTTTCAGCCAAAGCTGCAGTAGACTGCTCGGTTCCATTAAGCATAAGTGCCAGCATCAAATATGCAGACCAACAACGAAGAGAGAAACTTAAAAAGGAATTAGCACGATGTGAAAGGGAGTTGAAATTAAATAAAACAGCAATGCAGGCCAATTCTAAAAATAATTCCAAGTCATTATTTAACACTCTACAAAAGCCCTCAGGAGAACCACAAGATGAAGATGTGTTAATCAGAGAAGTGAATGGATTTCCATCCTTTACAAGGTCACCAGTAACTTCTTCAGAGAGACTACACCTAAATATACCTAAATCTGATAAAGTCCTCACAAATGGTACTGAGAAGAACTCCAGTCCCTGTCTGTTCAACGTGGATTACACCACCTCTGGGCCCAGGAGACCATGCTCTGGAACATCACATGGCCGAGGGCCCAAGAGCACGTTCCCAAATACGCATCGCTTTCAGTTAGTTATTTCAAAAGCACCCAGTGGGGACCTTTTGGATAAACATTCTGAACTCTTTTCCAACAGACATTTGCCATTCACTCCACGCACTTTAAAAACAGAAGCAAAGTCTTTCCTGTCACATTATCGATATTACACACCTGCCAGAAGAAAAAAGGATTTTACAGATCAGCGGATGGAAGCTGAAACCCAGACTGAATTAAGCAGCTTTAAATCTGATTTTGAGACAGTTGACACCAAGAACTTCACAGATTCAGAAGAGAACATAAAGCAGGCATCTAACTGTTTGACAGATGATACCAAAGGAAAAATAAATCCTTTACCCCTACAAGGGCATGACTTACCATGGGATGAGATCAAAGATGGCACTCTTCAGTGCTCCTCACCAAGGGCAGTATGTCAGTATTCCCTGCAGCTTCCTCCAGAGAGAAAAATCTACTCTGAGTAAGAGCTTTTTGACCTCTTTATTTTGCTTAGTGGA

***B. Predicted protein***

Peptide XP_038401425.1. Deleted fragment in red and underscored. AA change (Asp361Glu) highlighted.

>Predicted_SPATA7_Protein

MDGSRRVRATSVLPRYGTPCLFKGHLSTKSNAFCTDSSSLRLSTLHLVKNHMAVHYNKILSAKAAVDCSVPLSISASIKYADQQRREKLKKELARCERELKLNKTAMQANSKNNSKSLFNTLQKPSGEPQDEDVLIREVNGFPSFTRSPVTSSERLHLNIPKSDKVLTNGTEKNSSPCLFNVDYTTSGPRRPCSGTSHGRGPKSTFPNTHRFQLVISKAPSGDLLDKHSELFSNRHLPFTPRTLKTEAKSFLSHYRYYTPARRKKDFTDQRMEAETQTELSSFKSDFETVDTKNFTDSEENIKQASNCLTDDTKGKINPLPLQGHDLPWDEIKDGTLQCSSPRAVCQYSLQLPPERKIYSDEEELLYLSFIEDVTDEILKLGLFSNRFLERLFERHIKQNKHHLEEGKMRHLLHILKVDLGCTSRENSVKLDDLDMLDLHDFEQAENSKENEFRNKHDTTIQQERQEYQKALDMLLSIPKDENERLSSPNEFFLPVYKSKYSEGVIIQQVNDETNLGPSSWDEKNPSVSDSLTDQETSVNVIEGDSDSEKVEPSNELCCLSTELSPALQFHSVQGDNSHNMEGPTLKIMEMSIED

>Predicted_Canine_SPATA7_Protein_mutant

MDGSRRVRATSVLPRYGTPCLFKGHLSTKSNAFCTDSSSLRLSTLHLVKNHMAVHYNKILSAKAAVDCSVPLSISASIKYADQQRREKLKKELARCERELKLNKTAMQANSKNNSKSLFNTLQKPSGEPQDEDVLIREVNGFPSFTRSPVTSSERLHLNIPKSDKVLTNGTEKNSSPCLFNVDYTTSGPRRPCSGTSHGRGPKSTFPNTHRFQLVISKAPSGDLLDKHSELFSNRHLPFTPRTLKTEAKSFLSHYRYYTPARRKKDFTDQRMEAETQTELSSFKSDFETVDTKNFTDSEENIKQASNCLTDDTKGKINPLPLQGHDLPWDEIKDGTLQCSSPRAVCQYSLQLPPERKIYSE

***C. Anti-SPATA7 antibodies used***

Alignment of the epitopes against the protein. Segment lost in the mutant in red.

SPATA7: Canine SPATA7 protein sequenc. INTER: antibody NBPI-56884 epitope. CTERM: Antibody 12020-1-AP epitope. Note how both antibody have at least part of the epitope falling within the interval of the mutated protein.

SPATA7 MDGSRRVRATSVLPRYGTPCLFKGHLSTKSNAFCTDSSSLRLSTLHLVKNHMAVHYNKIL 60

INTER ------------------------------------------------------------ 0

CTERM ------------------------------------------------------------ 0

SPATA7 SAKAAVDCSVPLSISASIKYADQQRREKLKKELARCERELKLNKTAMQANSKNNSKSLFN 120

INTER ------------------------------------------------------------ 0

CTERM ------------------------------------------------------------ 0

SPATA7 TLQKPSGEPQDEDVLIREVNGFPSFTRSPVTSSERLHLNIPKSDKVLTNGTEKNSSPCLF 180

INTER ------------------------------------------------------------ 0

CTERM ------------------------------------------------------------ 0

SPATA7 NVDYTTSGPRRPCSGTSHGRGPKSTFPNTHRFQLVISKAPSGDLLDKHSELFSNRHLPFT 240

INTER ------------------------------------------------------------ 0

CTERM ------------------------------------------------------------ 0

SPATA7 PRTLKTEAKSFLSHYRYYTPARRKKDFTDQRMEAETQTELSSFKSDFETVDTKNFTDSEE 300

INTER ----------FLSQYRYYTPAKRKKDFTDQRIEAETQTEL-SFKSELGTAETKNMTDSEM 49

CTERM ---------SFLSQYRYYTPAKRKKDFTDQRIEAETQTEL-SFKSELGTAETKNMTDSEM 50

***:*******:*********:******** ****:: *.:***:****

SPATA7 NIKQASNCLTDDTKGKINPLPLQGHDLPWDEIKDGTLQCSSPRAVCQYSLQLPPERKIYS 360

INTER N----------------------------------------------------------- 50

CTERM NIKQASNCVTYDAKEKIAPLPLEGHDSTWDEIKDDALQHSSPRAMCQYSLKPPSTRKIYS 110

*

SPATA7 DEEELLYLSFIEDVTDEILKLGLFSNRFLERLFERHIKQNKHHLEEGKMRHLLHILKVDL 420

INTER ------------------------------------------------------------ 50

CTERM DEEELLYLSFIEDVTDEILKLGLFSNRFLERLFERHIKQNK-HLEEEKMRHLLHVLKVDL 169

SPATA7 GCTSRENSVKLDDLDMLDLHDFEQAENSKENEFRNKHDTTIQQERQEYQKALDMLLSIPK 480

INTER ------------------------------------------------------------ 50

CTERM GCTSEENSVKQNDVDMLNVFDFEKAGNSEPNELKNES-ETIQQERQQYQKALDMLLSAPK 228

SPATA7 DENERLSSPNEFFLPVYKSKYSEGVIIQQVNDETNLGPSSWDEKNPSVSDSLTDQETSVN 540

INTER ------------------------------------------------------------ 50

CTERM DENEIFPSPTEFFMPIYKSKHSEGVIIQQVNDETNLETSTLDENHPSISDSLTDRETSVN 288

SPATA7 VIEGDSDSEKVEPSNELCCLSTELSPALQFHSVQGDNSHNMEGPTLKIMEMSIED----- 595

INTER ------------------------------------------------------------ 50

CTERM VIEGDSDPEKVEISNGLCGLNTSPSQSVQFSSVKGDNNHDMELSTLKIMEMSIEDCPLDV 348
