# Supplementary material for: Two genes, one culprit - a functional candidate validation of a SPATA7 deletion in dogs with day blindness/retinal degeneration
Source: PLoS Genet. 2025 Dec 1;21(12):e1011961. doi: 10.1371/journal.pgen.1011961 (PMC12680346; doi:10.1371/journal.pgen.1011961)
Supplement: S3 File — References are also indicated in the main article [29,61,65,66,71–77]. (DOCX) [file pgen.1011961.s008.docx]

**S3 File. Supplementary Methods**

**Pedigree analysis**

Ancestry information on the cases was retrieved from the pedigrees accompanying clinical records/sample, and then searched in available Standard poodle databases (Standardpoodledatabase.com, accessed 08/03/2025; and Poodle.pedigreedatabaseonline.com, accessed 08/03/2025). Pedigree information was then parsed using a text editor and plotted using the R package kinship2 [71] in the R Studio integrated environment (http://www.rstudio.com/, accessed 08/03/2025) using default settings (**S2 Fig**). Due to the size and depth of the family tree and to exclude any possible instance of random shared ancestry of the cases, we selected 7 unrelated unaffected SPs from our clinical records and checked their ancestry to see if they shared the same common ancestor as the cases; the results were negative.

### Estimation of the age of the mutation

This estimate is based on the method described by Gandolfo and colleagues [72] which estimates the age of a genetic variant based on the length of the ancestral haplotypes shared between the individuals carrying the causative variant. The website (https://shiny.wehi.edu.au/rafehi.h/mutation-dating/, accessed 08/03/2025) with the “advanced user - input text all species” parameter and the default 0.95 confidence interval was used for these calculations.

***RNA transcript analysis***

Dog skin biopsies as to obtain primary skin fibroblasts, were lysed with high impact zirconium beads and trizol for RNA extraction and subsequent cDNA synthesis. Primers were designed to amplify three different parts of the transcript (central, closer to 5’, closer to 3’). Part of the RNA was used for the preparation of standard RNA-seq libraries.

**Fibroblast isolation for RNA-seq analysis**

Dog skin biopsies were dissociated as previously described to obtain primary skin fibroblasts [73]. For immunocytochemistry, fibroblasts preparation followed a different protocol described below. The entirety of the extracted fibroblast samples was used for the RNA extraction. It was transferred into a 2 mL tube containing 3.0 mm high impact zirconium beads (Prefilled Bead Bug Tubes, Benchmark D1032-30). The samples were homogenized in 1 mL of Trizol (Invitrogen) in the Digital Cell Disruptor Genie homogenizer (Scientific Industries) for 3x6 min at 3000 rpm at +4^o^C and incubated for 5 min. The homogenate was transferred into a new tube and 200 µL chloroform was added for a 3-phase separation. The upper aqueous phase was transferred into a new tube, 75% EtOH was added to the sample, and gently mixed by flicking and up to 700 µL was transferred into the RNAeasy (Qiagen 74104) spin column in a collection tube from the same kit. The rest of the washing and elution passages followed the recommendation of the manufacturer. RNA concentrations were measured using NanoDrop and 1 ul RNaseOUT™ Recombinant Ribonuclease Inhibitor (Thermo Fisher) was added 1:10. SuperScript VILO cDNA synthesis kit (Invitrogen 11754050) was then used to synthesize the cDNA, following manufacturer's instructions (1 hr amplification at 42°C followed by 5 minutes of 85°C for enzyme inactivation).

*Mutant transcript amplification*

The impact of the *SPATA7* homozygous mutant transcript deletion was verified in the fibroblast cell culture of one control, one case and one carrier by re-sequencing of PCR products by Sanger sequencing. PCR primers were designed using PRIMER3 [64], and products were run on 1.5% agarose gel and 0.5 μg/mL ethidium bromide. PCR products were amplified using primers targeting different exons. Amplicon A, 230 bp, F: GCACTGACTCCTCCTCTCTC (Exon 3), R: ATTGGCCTGCATTGCTGTTT (Exon 5); amplicon B, 252 bp, F: GCGGATGGAAGCTGAAACC (Exon 6), R: GGAGGAAGCTGCAGGGAATA (Exon 9); amplicon C, 221 bp, F: ACGACTGTTTGAGCGACATA (Exon 11), R: CCTGTTGAATTGTGGTGTCGT (Exon 12). All primers are written 5’-3’. All PCRs were amplified with AmpliTaqGold360Mastermix (Life Technologies). The products and the boundaries of the deletion were verified by Sanger sequencing. Sequence data were visualized using 4Peaks v 1.8.

**RNA-sequencing**

Additional extracted fibroblast RNA was used to produce three RNA-seq libraries that were sequenced using the Illumina Novaseq 6000. One library consisted of RNA from a case, another from a carrier and another from a homozygous wild-type SPATA7 variant. After quality control, RNA-seq reads were aligned to the canine reference (canFam4) using STAR v2.7.0 [74], according to the standard parameters. Aligned reads were visually compared among themselves and against the deletion genetic variant, and the missing exons were also visually confirmed, using the Integrative Genomics Viewer [75].

**Canine skin fibroblast isolation and culture**

The dissociation of canine skin biopsies, culture of skin fibroblasts, and induction of ciliogenesis were conducted following previously described protocols [76]. In brief, skin biopsies were dissociated by incubation in a solution containing 5 U/mL dispase and 1mg/mL collagenase type IV for 3 hours, followed by filtration through a 100 μm cell strainer [76]. Following centrifugation at 300 x g for 8 minutes, the cells were cultured in DMEM/F-12 supplemented with 10% FBS and supplements [76]. Once passaged, the cells were seeded on glass coverslips and incubated in 10% FBS containing medium for 24 hours. Subsequently, the cells were cultured in serum-free medium for 48 hours to induce ciliogenesis. The ciliated cells were fixed with cold methanol at -20°C for 8 minutes and then immunohistochemistry was performed on both non-expanded conventional cells and cells treated for ultrastructure expansion microscopy (U-ExM).

**Retinal Tissue Processing**

The procedures for cryopreservation of normal adult canine ocular tissues were performed as described previously [77]. Briefly, the posterior eyecups were immersion-fixed in 4% paraformaldehyde (PFA) in 0.1 M phosphate-buffered saline (PBS) for 3 hours, followed by an additional 24-hour fixation in 2% PFA at 4 °C. The tissues were then cryoprotected sequentially in 15% and 30% sucrose, embedded in optimal cutting temperature (OCT) compound, and rapidly frozen in liquid nitrogen–cooled methylbutane. In addition, the eyes from one affected dog (SP29) and one control (EM540) were fixed in modified Davidson’s fixative for over 24 hours and processed for paraffin embedding.

**Ultrastructure expansion microscopy (U-ExM) and immunolabeling**

The U-ExM procedure for skin fibroblasts was performed following an optimized protocol for visualizing the primary cilium, as described previously [65]. U-ExM on retinal tissue was conducted according to a published protocol [29]. Sections from paraffin-embedded tissues were deparaffinized using xylene and ethanol before U-ExM processing. For immunostaining, the following primary and secondary antibodies were used: anti-acetylated tubulin (mouse, Sigma-Aldrich T7451, 1:1000), anti-Centrin (mouse, Sigma-Aldrich 04-1624, 1:200), anti-PTPN21 (rabbit, Bioss Antibodies BS-19587R, 1:200), anti-PTPN21 (rabbit, Sigma-Aldrich T5953, 1:200), anti-SPATA7 [C-term] (rabbit, Proteintech 12020-1-AP, 1:200), anti-Rhodopsin (mouse, Sigma-Aldrich MAB5316, 1:1000), anti-SPATA7 [inter] (rabbit, Novus Biologicals NBP1-56884, 1:200), Alexa Fluor 488 conjugated secondary antibody (made in goat, Thermo Fisher Scientific A21141, 1:1000), Alexa Fluor 568 conjugated secondary antibody (made in goat, Thermo Fisher Scientific A21121 and A21134, 1:1000), and Alexa Fluor 647 conjugated secondary antibody (made in goat, Thermo Fisher Scientific A21246, 1:1000). Image acquisition was conducted using the Leica Stellaris 8 FALCON confocal FLIM microscope equipped with 100x oil (1.40 NA) and 63x water (1.20 NA) objectives.

**Real-time PCR**

Quantitative real-time PCR was performed on an Applied Byosystems on a 96-well plate. SYBR Green kit and gene-specific primers (Exons 8 and 9 amplicon F: CCCTACAAGGGCATG ACTTAC, R: GAGGAAGCTGCAGGGAATAC; Exon 12 F: CCCAGCTCTTCAGTTTCA CA R: CTAAGGTCAGTCCTCAATGCTC) were used. The reaction was used determine whether the expression of the wild-type and the mutant transcript was significantly different between two cases (SP17 and SP20, three technical replicates each) and two controls (N365 and E1075, three technical replicates each). Normalization was performed on the Ct scale by computing $\Delta C_{t}=C_{t,target}-C_{t,GAPDH}$, where $C_{t,GAPDH}$is the sample-level mean GAPDH Ct across its triplicates. Set 2 ΔCt values were compared between the cDNA amplification of the affected SPs and the controls using Welch’s t-test.

**References**

29. Takahashi K, Sudharsan R, Beltran WA. Mapping Protein Distribution in the Canine Photoreceptor Sensory Cilium and Calyceal Processes by Ultrastructure Expansion Microscopy. Invest Ophthalmol Vis Sci. 2025;66(2):1. Epub 2025/02/03. doi: 10.1167/iovs.66.2.1. PubMed PMID: 39898911; PubMed Central PMCID: PMCPMC11798334.

61. Robinson JT, Thorvaldsdottir H, Wenger AM, Zehir A, Mesirov JP. Variant Review with the Integrative Genomics Viewer. Cancer Res. 2017;77(21):e31-e4. Epub 2017/11/03. doi: 10.1158/0008-5472.CAN-17-0337. PubMed PMID: 29092934; PubMed Central PMCID: PMCPMC5678989.

64. Untergasser A, Cutcutache I, Koressaar T, Ye J, Faircloth BC, Remm M, et al. Primer3—new capabilities and interfaces. Nucleic Acids Research. 2012;40(15):e115-e. doi: 10.1093/nar/gks596.

65. Gambarotto D, Hamel V, Guichard P. Ultrastructure expansion microscopy (U-ExM). Methods Cell Biol. 2021;161:57-81. Epub 2021/01/23. doi: 10.1016/bs.mcb.2020.05.006. PubMed PMID: 33478697.

71. Sinnwell JP, Therneau TM, Schaid DJ. The kinship2 R package for pedigree data. Hum Hered. 2014;78(2):91-3. Epub 2014/07/31. doi: 10.1159/000363105. PubMed PMID: 25074474; PubMed Central PMCID: PMCPMC4154601.

72. Gandolfo LC, Bahlo M, Speed TP. Dating rare mutations from small samples with dense marker data. Genetics. 2014;197(4):1315-27. Epub 2014/06/01. doi: 10.1534/genetics.114.164616. PubMed PMID: 24879464; PubMed Central PMCID: PMCPMC4125402.

73. Shimada H, Lu Q, Insinna-Kettenhofen C, Nagashima K, English MA, Semler EM, et al. In Vitro Modeling Using Ciliopathy-Patient-Derived Cells Reveals Distinct Cilia Dysfunctions Caused by CEP290 Mutations. Cell Rep. 2017;20(2):384-96. Epub 2017/07/13. doi: 10.1016/j.celrep.2017.06.045. PubMed PMID: 28700940; PubMed Central PMCID: PMCPMC5553702.

74. Dobin A, Davis CA, Schlesinger F, Drenkow J, Zaleski C, Jha S, et al. STAR: ultrafast universal RNA-seq aligner. Bioinformatics. 2013;29(1):15-21. doi: 10.1093/bioinformatics/bts635. PubMed PMID: WOS:000312654600003.

75. Thorvaldsdottir H, Robinson JT, Mesirov JP. Integrative Genomics Viewer (IGV): high-performance genomics data visualization and exploration. Brief Bioinform. 2013;14(2):178-92. Epub 2012/04/21. doi: 10.1093/bib/bbs017. PubMed PMID: 22517427; PubMed Central PMCID: PMCPMC3603213.

76. Takahashi K, Kwok JC, Sato Y, Aguirre GD, Miyadera K. Molecular characterization of MAP9 in the photoreceptor sensory cilia as a modifier in canine RPGRIP1-associated cone-rod dystrophy. Front Cell Neurosci. 2023;17:1226603. Epub 2023/08/31. doi: 10.3389/fncel.2023.1226603. PubMed PMID: 37650070; PubMed Central PMCID: PMCPMC10464610.

77. Takahashi K, Beltran WA, Sudharsan R. An optimized workflow for transcriptomic analysis from archival paraformaldehyde-fixed retinal tissues collected by laser capture microdissection. Exp Eye Res. 2024;246:109989. Epub 2024/07/06. doi: 10.1016/j.exer.2024.109989. PubMed PMID: 38969282; PubMed Central PMCID: PMCPMC11330715.
